# Genome-wide characterization and phylogenetic analysis of *GSK* gene family in three species of cotton: evidence for a role of some *GSKs* in fiber development and responses to stress

**DOI:** 10.1186/s12870-018-1526-8

**Published:** 2018-12-04

**Authors:** Lingling Wang, Zhaoen Yang, Bin Zhang, Daoqian Yu, Ji Liu, Qian Gong, Ghulam Qanmber, Yi Li, Lili Lu, Yongjun Lin, Zuoren Yang, Fuguang Li

**Affiliations:** 1 State Key Laboratory of Cotton Biology, Key Laboratory of Biological and Genetic Breeding of Cotton, Institute of Cotton Research, Chinese Academy of Agricultural Sciences, Anyang, 455000 Henan China; 2 National Key Laboratory of Crop Genetic Improvement and National Centre of Plant Gene Research, Huazhong Agricultural University, Wuhan, 430070 People’s Republic of China

**Keywords:** GSK3 (glycogen synthase kinase 3/shaggy kinase), Cotton, Genome duplication, Fiber development, Abiotic stress, Co-expression network

## Abstract

**Background:**

The glycogen synthase kinase 3/shaggy kinase (GSK3) is a serine/threonine kinase with important roles in animals. Although *GSK3* genes have been studied for more than 30 years, plant *GSK* genes have been studied only since the last decade. Previous research has confirmed that plant *GSK* genes are involved in diverse processes, including floral development, brassinosteroid signaling, and responses to abiotic stresses.

**Result:**

In this study, 20, 15 (including 5 different transcripts) and 10 *GSK* genes were identified in *G. hirsutum*, *G. raimondii* and *G. arboreum*, respectively. A total of 65 genes from *Arabidopsis*, rice, and cotton were classified into 4 clades. High similarities were found in GSK3 protein sequences, conserved motifs, and gene structures, as well as good concordance in gene pairwise comparisons (*G. hirsutum* vs. *G. arboreum*, *G. hirsutum* vs. *G. raimondii*, and *G. arboreum* vs. *G. raimondii*) were observed. Whole genome duplication (WGD) within At and Dt sub-genomes has been central to the expansion of the *GSK* gene family. Furthermore, *GhSK* genes showed diverse expression patterns in various tissues. Additionally, the expression profiles of *GhSKs* under different stress treatments demonstrated that many are stress-responsive genes. However, none were induced by brassinolide treatment. Finally, nine co-expression sub-networks were observed for *GhSKs* and the functional annotations of these genes suggested that some *GhSK*s might be involved in cotton fiber development.

**Conclusion:**

In this present work, we identified 45 *GSK* genes from three cotton species, which were divided into four clades. The gene features, muti-alignment, conversed motifs, and syntenic blocks indicate that they have been highly conserved during evolution. Whole genome duplication was determined to be the dominant factor for *GSK* gene family expansion. The analysis of co-expressed sub-networks and tissue-specific expression profiles suggested functions of *GhSKs* during fiber development. Moreover, their different responses to various abiotic stresses indicated great functional diversity amongst the *GhSKs*. Briefly, data presented herein may serve as the basis for future functional studies of GhSKs.

**Electronic supplementary material:**

The online version of this article (10.1186/s12870-018-1526-8) contains supplementary material, which is available to authorized users.

## Background

Glycogen synthase kinase 3 (GSK3), which is also known as the SHAGGY-like protein kinase, is a non-receptor serine/threonine protein kinase and involved in vital signal transduction pathways in eukaryotes [1, 2]. In mammals, GSK3 exists as two isoforms, namely GSK3α and GSK3β, both of which help to regulate glycogen metabolism [3]. GSK3 was first reported as an enzyme-inactivating kinase in rabbit skeletal muscle [4]. The products of GSK3 homologs were confirmed to be involved in several physiological processes in animals, including protein synthesis, glycogen metabolism, regulation of transcription factor activity, determination of cell fate, and tumorigenesis [5–8]. Additionally, GSK3 is a key component of the animal Wnt signaling pathway [9, 10].

The plant *GSK3* genes appear to be more diverse than the corresponding animal genes, as evidenced by the fact that there are ten *Arabidopsis thaliana* genes and nine rice genes [1]. The plant *GSK3* genes were as diverse in function as animal isoforms. Biochemical and genetic analyses demonstrated that different plant GSK3 members affect disparate processes, such as development, stress responses, and signaling pathways, by phosphorylation of different protein substrates. In *A. thaliana*, *AtSK11* and *AtSK12* were highly expressed during the early stages of differentiation of the floral primordium. These two genes were subsequently expressed in specific regions, including the pollen-containing area of the anther, carpels, petals, and sepal primordial cells [11]. Previous studies revealed that *AtSK31*, which was localized mainly to the nuclei of developing tissues, was highly expressed in floral organs [12, 13]. Moreover, overexpression of different *AtSK32* isoforms in *A. thaliana* resulted in different phenotypes. Two mutations of AtSK32, Lys167, and Arg178 (homologous to the critical active site residues Lys85 and Arg96 of mammal GSK3β) were generated by site-directed mutagenesis [14]. However, overexpression of wild-type or catalytically inactive mutant (i.e., encoding a K167A mutation) *AtSK32* gene in *A. thaliana* produced no observable effects. Nevertheless, overexpression of *AtSK32*-*R178A* displayed shorter pedicels and smaller petals compared with wild-type controls [14]. Moreover, ASKα/AtSK11, which was an *A. thaliana* GSK3, regulated stress resistance by activating Glc-6-phosphate dehydrogenase (G6PD). Increased G6PD activity accompanied with decreased reactive oxygen species levels have been observed in *ASKα*-overexpressing plants under stress conditions, especially during salt stress [15]. GSK3/SHAGGY-like kinase (AtSK21) gain-of-function mutations or over-expressing transgenetic lines inhibited the brassinosteroid (BR) signaling pathway and led to BR-deficiency and suppression of BR-induced responses [16]. Upregulating the expression level of *OsGSK3* in rice (*Oryza sativa L.* ‘Nipponbare’) enhanced its tolerance to salt, cold, drought and mechanical injury, and abscisic acid (ABA) responsiveness [6]. There are ongoing studies aimed at comprehensively examining *GSK3* family members in various plant species, even though the number of putative *GSK3* genes is relatively low.

Cotton fibers, which are the premier natural fiber derived from the seedcoat epidermal cells, are the most important renewable resource used by the textile industry. The genus *Gossypium* that includes approximately 50 species, of which four are cultivated for their cotton fibers (i.e., *G. arboreum* and *G. herbaceum*, 2n = 2X = AA = 26; *G. hirsutum* (upland cotton), and *G. barbadense* (sea island cotton), 2n = 4X = AADD = 52) [17]. *Gossypium raimondii*, another diploid, carries a D-genome and only produces very short and coarse seed fibers [17]. *Gossypium hirsutum* is the most widely cultivated cotton species and accounts for more than 90% of the global cotton fiber yield [18]. This allotetraploid species contains two different sets of chromosomes (i.e., A and D) evolved as a result of inter-specific hybridization during the Pleistocene about 1–2 million years ago [17, 19]. Cotton fiber development is a complicated process involving several phytohormones, including auxin, ethylene, gibberellins (GAs), and BRs [20–25]. The BIN2 protein, as a negative regulator of BR signaling pathway, was a well-characterized GSK3 that could affect the activities of phytohormone-signaling pathways [26–32]. Additionally, the complete genome sequencing of *G. hirsutum*, *G. arboreum* and *G. raimondii* now allow genome-wide analyses of any gene family in cotton [19, 33–36]. However, there have been no systematic investigations of the *GSK* gene family from these three cotton species. Considering the functional importance of the *GSK* gene family, we conducted an in silico genome-wide search and analysis to identify and characterize the *GSK* gene family members of *G. arboreum*, *G. hirsutum*, and *G. raimondii*. We subsequently analyzed a multi-sequence alignment, gene loci, gene structures, promoter *cis*-elements, conserved protein motifs, phylogenetic relationships, gene expression profiles, and a weighted gene co-expression sub-network analysis. The results described herein may be useful for further functional characterizations of cotton GSKs. Our data may also help to clarify the mechanism underlying the regulatory effects of GSKs during cotton development, growth, and responses to stress conditions.

## Methods

### Plant materials and growth conditions

*Gossypium hirsutum* L. ‘*cv CCRI24*’ plants were grown in mixed soil under glasshouse conditions [14 h light (28~ 34 °C)/ 10 h dark (24~ 27 °C); 150 μmol m^− 2^ s^− 1^]. To analyze organ- and tissue-specific gene expression patterns in ‘CCRI24’, plants were grown under field conditions in Zhengzhou, China following standard crop management practices. Flower (whole flower at 0 dpa) and ovule samples were collected at 1, 3, 5 days post-anthesis (dpa). Additionally, isolated fibers were collected at 7, 10, 15, and 20 dpa. Stages of the tissues collected were chosen as described in previously published research [34, 37]. Three biological replicates were collected for each sample. All collected samples were immediately frozen in the liquid nitrogen and stored at − 80 °C for RNA extraction and subsequent analysis.

### Abiotic stress assays and BL treatment

The expression patterns of *GhSK* genes in response to various environmental stresses and brassinolide treatment were analyzed. For abiotic stresses, phenotypically similar *G. hirsutum* potted seedlings grown in a glasshouse up to the three-true-leaves stage (four weeks old) were selected [38, 39]. Each treatment consisted of three biological replicates of the individual seedling. For brassinolide treatment, seedlings were cultured in deionized water supplemented with 10 μM brassinolide, and leaf samples were collected at 0, 0.5, 1, 3, 5 h. The cold and heat treatments involved incubations at 4 and 38 °C, respectively. To simulate dehydration stress, cotton seedlings were irrigated with 20% polyethylene glycol (PEG) instead of water. Additionally, cotton seedling roots were immersed in 300 mM NaCl solutions to assess the effects of salt stress. The true leaves were collected at 1, 3, 6, and 12 h for all of the abiotic stresses performed as described in previously published investigations [38, 39]. All collected leaf samples were immediately frozen in liquid nitrogen and stored at − 80 °C for subsequent RNA isolation and cDNA synthesis.

### RNA isolation and quantitative real-time polymerase chain reaction analysis

Total RNA was extracted from collected cotton tissues/organs using the RNAprep Pure Plant Kit (TIANGEN, Beijing, China). First-strand cDNA was synthesized using the PrimeScript™ RT Reagent Kit, during which the genomic DNA was eliminated with gDNA Eraser (Perfect Real Time; Takara, Dalian, China). The quantitative real-time polymerase chain reaction (qRT-PCR) primers were designed by the Primer Premier 5.0 software. *Histone 3* (GenBank accession no. AF024716) was selected as the reference gene. The qRT-PCR was conducted using SYBR Premix Ex Taq™ (Tli RNase H Plus) (Takara) and ABI 7900 qRT-PCR System (Applied Biosystems, CA, USA). The PCR program was as follows: 95 °C for 30 s; 40 cycles of 95 °C for 5 s and 60 °C for 20 s. The 2^−ΔΔCt^ method was applied to calculate the relative expression level of all target genes as compared to control treatments [40].

### Identification of cotton *GSK* genes

The latest version of the *A. thaliana* and rice genome, protein sequence and annotation databases were downloaded at http://www.arabidopsis.org/ and http://plants.ensembl.org/index.html [41], respectively. Additionally, the genome sequence versions of the *G. hirsutum* (NAU, Version 1.1) [34], *G. raimondii* (JGI, version2.0) [19] available from COTTONGEN (https://www.cottongen.org) [42] and *G. arboreum* (PacBio-Gar-Assembly-v1.0, ftp://bioinfo.ayit.edu.cn/downloads/) [43] were used to identify GSK proteins and their corresponding nucleotide sequences. The cotton *GSK* genes and encoded proteins were identified via a BLAST search using all of the *A. thaliana ASK* gene and encoded protein sequences as queries. For protein analyses, the following parameters were used: e-value = 1e-5 and coverage ratio = 50%. Moreover, the definition of the Pkinase (PF00069.23) domain was downloaded from Pfam: http://pfam.janelia.org/ [44], and then the hidden Markov model (HMM) was used to verify the GSKs from the three cotton species. The identified *A. thaliana*, rice, *G. hirsutum*, *G. arboreum*, and *G. raimondii* genes were renamed as previously reported [45] based on their order of phylogenetic clustering.

### Multiple-sequence alignment and phylogenetic analysis

A sequence alignment of full-length protein sequences from the three analyzed cotton species, *A. thaliana*, and rice was prepared using MUSCLE: http://www.ebi.ac.uk/Tools/msa/muscle/ and saved in the ClustalW format. Meanwhile, a phylogenetic analysis of the *A. thaliana*, rice, and cotton GSKs was conducted using the MEGA 6.0 program, with 1000 bootstrap replications [46]. An unrooted Neighbor-joining, as well as Minimum-Evolution tree were constructed using the Poisson model method using the same alignment file.

### Comparison of chromosomal distributions, exon/ intron structures, and protein domains among a, D, and AD cotton genomes

The genomic distribution of the cotton *GSK* genes was analyzed by MapInspect software (https://mapinspect1.software.informer.com/) according to the start positions indicated in *G. hirsutum*, *G. arboreum*, and *G.raimondii* databases [47]. The intron/ exon structures were examined using the Gene Structure Display Server 2.0 program (http:/gsds.cbi.pku.edu.cn/). Meanwhile, the cotton GSK domains were predicted with the MEME (Multiple Expectation Maximization for Motif Elicitation) online tool: http://meme-suite.org/tools/meme [48] using the following parameters: motif width 6–200 residues and the maximum number of motifs = 20. The mast.xml file exported from MEME was used to generate the motif images using TBtools_master (version 0.49991) (https://github.com/CJ-Chen/TBtools). The conserved motif logos were downloaded from MEME as well.

### Synteny and gene duplications analysis

The *G. hirsutum*, *G. arboreum*, and *G. raimondii* genome data were searched using a BLAST-Like Alignment Tool (BLAT) [49] to identify tandem duplications which were defined as multilocus genes located in adjacent regions or separated by uniform intergenic regions. Sequences with coverage > 90% and similarity > 95% were designated as tandem duplicates.

We performed the synteny analysis of *GSK* genes among the three cotton species included in this study (i.e., *G. hirsutum* vs. *G. arboreum*, *G. hirsutum* vs. *G. raimondii*, and *G. arboreum* vs. *G. raimondii*). BLAT was used for the pairwise comparison of *G. hirsutum*, *G. arboreum*, and *G. raimondii* gene sets and to identify the homologous genomic regions. Finally, the syntenic blocks were visualized using the default parameters of the Circos (version 0.69) program [50].

### Analysis of the promoter cis-regulatory elements

The 2-kb sequences upstream of the cotton *GhSK* genes (i.e., putative promoter regions) were obtained by BLAST searches of the cotton genome data using whole gene IDs. Potential *cis*-acting regulatory elements of the extracted sequences were subsequently subjected to PlantCARE database analysis (http://bioinformatics.psb.ugent.be/webtools/plantcare/html/) [51]. The identified *cis*-elements were drawn using a custom script in the R program (version 3.20; https://www.r-project.org/).

### Biophysical properties of *GSK* genes from three cotton species

The three genome sets of cotton GSK protein sequences were analyzed using ExPASy-ProtParam tool: http://web.expasy.org/protparam/ to calculate the number of amino acids, molecular weight, and theoretical pI [52]. Meanwhile, the subcellular localization of these genes was predicted with ProComp 9.0 (http://www.softberry.com/berry.phtml?group=programs&subgroup=proloc&topic=protcomppl) [53].

### Weighted co-expression sub-network analysis of *GhSK* genes

The weighted gene co-expression sub-network analysis (WGCNA) of the *GhSK* genes was conducted using the *G. hirsutum* TM-1 transcriptome sequencing data downloaded from the NCBI Sequence Read Archive database [34, 54]. The network construction and module detection were performed using the ‘cuttreeDynamic’ and ‘mergeCloseModules’ by “WGCNA” R package (version 1.4.9) [55], the parameters were set as follows: The power was 9; the minModuleSize was 30 and the cutHeight was 0.25. Finally, we visualized the sub-network using Cytoscape (version 3.4.0) program [56]. An additional investigation of the putative functions of *GhSK* genes and their co-expression genes was based on Gene Ontology (GO) and Kyoto Encyclopedia of Genes and Genomes (KEGG) enrichment analyses.

### Gene expression patterns analyzed using published transcriptomic data

The high-throughput *G. hirsutum* TM-1 transcriptome sequencing data were used to investigate the *GhSK* gene expression patterns in vegetative tissues, fiber tissues, floral organs, and dry seed. The log10 transformed Fragments Per Kilobase of transcript per Million fragments (FPKM) mapped values of the 20 *GhSK* genes were calculated to generate heatmaps with the local Multiple Arrays Viewer program (http://www.mybiosoftware.com/mev-4-6-2-multiple-experiment-viewer.html). The accession numbers and related sample information of the data used in this study are listed in Additional file 1: Table S1.1, S1.2 and Additional file 2: Table S2.

### Statistical analysis

Data analyses here were executed through one-way analysis of variance (ANOVA) and a Dunnett’s test at *p* < 0.05 was performed. Taking the biological significance of the differential expression into account, we assumed a two-fold cut-off value for analyzing the stress and hormone induction or inhibition [57]. The expression levels were designated as ‘induced’ or ‘inhibited’ only when the differences met the specified criteria.

## Results

### Genome-wide characterization of cotton *GSK* genes

After removing redundant sequences, 20, 10, and 15 *GSK* genes were identified in *G. hirsutum*, *G. arboreum*, and *G. ramondii* genomes, respectively. To avoid the possibility of confusion and overlap with the gene names used, we renamed these genes as *GhSKs*, *GaSKs*, and *GrSKs*. The genes were numbered sequentially according to the subfamilies to which they were assigned after phylogenetic analysis (Table 1).

**Table 1:** Characteristics of the putative cotton *GSK* genes

Subfamily	Gene ID	Proposed name	Amino acid length	MW (KDa)	PI	Subcellular location	Location
Clade I	Gh_D11G2830	GhSK11	407	46.20	8.59	Cyto_Nucl_Memb	D24: 58071261–58,074,075(−)
	Gh_A11G3270	GhSK12	407	46.20	8.59	Cyto_Nucl_Memb	Scaf3045_A11: 160892–163,705(−)
	Gh_A12G1106	GhSK13	409	46.41	8.69	Cyto_Nucl_Memb	A12: 64114146–64,116,909(+)
	Gh_D12G1230	GhSK14	409	46.31	8.58	Cyto_Nucl_Memb	D25: 40170076–40,172,846(+)
	Ga11G0763	GaSK11	407	46.23	8.59	Cyto_Nucl_Memb	Ga11:13088074–13,090,884(+)
	Ga12G1683	GaSK12	409	46.37	8.58	Cyto_Nucl_Memb	Ga12:26213850–26,216,616(+)
	Gorai.007G308500.1	GrSK11	407	46.20	8.59	Cyto_Nucl_Memb	Gr07:52192786–52,197,103(−)
	Gorai.007G308500.3	GrSK11–1	407	46.20	8.59	Cyto_Nucl_Memb	Gr07:52192786–52,197,103(−)
	Gorai.008G136600.1	GrSK12	409	46.36	8.58	Cyto_Nucl_Memb	Gr08:38585110–38,589,107(+)
Clade II	Gh_D06G2142	GhSK21	381	43.24	8.74	Cyto_Nucl_Memb	D19:63261021–63,263,915(−)
	Gh_D09G2469	GhSK22	382	43.10	8.74	Cyto_Nucl_Memb	Scaf4332_D22:137949–141,233(−)
	Gh_D09G2468	GhSK23	381	43.38	8.83	Cyto_Nucl_Memb	Scaf4332_D22:123124–126,295(−)
	Gh_A09G0713	GhSK24	381	43.32	9.02	Cyto_Nucl_Memb	A09:52861382–52,864,569(+)
	Gh_A09G0712	GhSK25	376	42.64	8.88	Cyto_Nucl_Memb	A09:52841598–52,844,894(+)
	Gh_A06G2020	GhSK26	385	43.72	8.69	Cyto_Nucl_Memb	Scaf1340_A06:43052–45,877(+)
	Ga06G2433	GaSK21	381	43.24	8.74	Cyto_Nucl_Memb	Ga06:130210718–130,213,627(+)
	Ga09G0899	GaSK22	376	42.59	8.84	Cyto_Nucl_Memb	Ga09:59971361–59,974,663(+)
	Ga09G0900	GaSK23	382	43.55	8.89	Cytoplasmic	Ga09:59988594–59,991,790(+)
	Gorai.010G241600.3	GrSK21	381	43.24	8.74	Cyto_Nucl_Memb	Gr10:61087060–61,090,705(−)
	Gorai.010G241600.2	GrSK21–1	381	43.24	8.74	Cyto_Nucl_Memb	Gr10:61085419–61,090,069(−)
	Gorai.006G089300.1	GrSK22	382	43.22	8.74	Cyto_Nucl_Memb	Gr06:32530678–32,535,037(+)
	Gorai.006G089300.2	GrSK22–1	381	43.10	8.74	Cyto_Nucl_Memb	Gr06:32530521–32,535,037(+)
	Gorai.006G089400.1	GrSK23	381	43.34	8.74	Cyto_Nucl_Memb	Gr06:32546669–32,551,023(+)
Clade III	Gh_A08G0285	GhSK31	469	52.95	8.75	Cyto_Nucl_Memb	A08:3329052–3,334,479(−)
	Gh_D08G1440	GhSK32	471	53.14	8.38	Cyto_Nucl_Memb	D21:47361120–47,366,590(+)
	Gh_D11G0907	GhSK33	470	53.00	7.27	Cyto_Nucl_Memb	D24:7839230–7,844,650(+)
	Gh_A08G1158	GhSK34	471	53.06	8.2	Cyto_Nucl_Memb	A08:81021333–81,025,764(+)
	Gh_A11G0778	GhSK35	470	52.87	6.81	Cyto_Nucl_Memb	A11:7649123–7,654,545(+)
	Gh_D08G0378	GhSK36	494	55.85	8.99	Cyto_Nucl_Memb	D21:3871236–3,880,133(−)
	Ga08G0389	GaSK31	469	52.95	8.2	Cyto_Nucl_Memb	Ga08:4054818–4,060,304(−)
	Ga11G3164	GaSK32	470	52.88	7	Cyto_Nucl_Memb	Ga11:116299523–116,304,934(−)
	Ga08G1552	GaSK33	471	53.10	8.2	Cyto_Nucl_Memb	Ga08:103960877–103,965,308(+)
	Gorai.004G042400.1	GrSK31	469	52.97	8.86	Cyto_Nucl_Memb	Gr04:3659148–3,665,477(−)
	Gorai.007G096100.1	GrSK32	470	53.04	7.6	Cyto_Nucl_Memb	Gr07:7076952–7,083,244(+)
	Gorai.004G156700.2	GrSK33	415	47.04	8.21	Cyto_Nucl_Memb	Gr04:44445510–44,453,846(+)
Clade IV	Gh_A01G1558	GhSK41	422	47.85	8.63	Cyto_Nucl_Memb	A01:92410497–92,413,873(+)
	Gh_D01G1809	GhSK42	422	47.85	8.63	Cyto_Nucl_Memb	D14:55472543–55,475,936(+)
	Gh_D12G0407	GhSK43	422	47.90	8.49	Cyto_Nucl_Memb	D25:6533937–6,537,332(−)
	Gh_A12G0411	GhSK44	422	47.87	8.49	Cyto_Nucl_Memb	A12:8244999–8,248,430(−)
	Ga02G1328	GaSK41	422	47.84	8.63	Cyto_Nucl_Memb	Ga02:91572951–91,576,357(+)
	Ga12G2665	GaSK42	439	50.02	8.68	Cyto_Nucl_Memb	Ga12:98904726–98,909,799(−)
	Gorai.002G218100.1	GrSK41	422	47.85	8.63	Cyto_Nucl_Memb	Gr02:56980865–56,985,951(+)
	Gorai.008G045800.6	GrSK42	422	47.89	8.49	Cyto_Nucl_Memb	Gr08:6198704–6,202,924(−)
	Gorai.008G045800.3	GrSK42–1	422	47.89	8.49	Cyto_Nucl_Memb	Gr08:6198649–6,203,828(−)
	Gorai.008G045800.2	GrSK42–2	422	47.89	8.49	Cyto_Nucl_Memb	Gr08:6198649–6,203,828(−)

The biophysical characteristics of the identified cotton GSKs are provided in Table 1. The number of amino acids of putative GhSK, GaSK, and GrSK proteins varied from 376 (GhSK25 and GaSK22) to 494 (GhSK36), and the associated molecular weights ranged from 42.59 to 53.14 kDa respectively. All pIs of the GSKs were higher than 7, except GhSK35 (6.81). Interestingly, the predicted subcellular localization revealed that almost all of the proteins were localized to multiple compartments including the cytoplasm, nucleus, and membrane, excepting for GaSK23 (only cytoplasmic). The localization results for the cotton GSKs were not the same as those that have been reported for *Arabidopsis* GSKs which might be because the cotton GSKs are much less well studied and their functions remain largely unknown.

### Phylogenetic analysis amongst the *Arabidopsis thaliana*, rice and cotton *GSK* genes

To reveal the phylogenetic relationships among the *GSK* genes, full-length *A. thaliana*, rice, and cotton protein sequences were aligned. The aligned GSKs were highly similar and the conserved GSK protein kinase region is presented in detail in Additional file 3: Figure S1. Additionally, the kinase domain [2] and the tyrosine residue essential for kinase activity [2] were conserved among all the cotton GSKs.

**Fig. 1:**
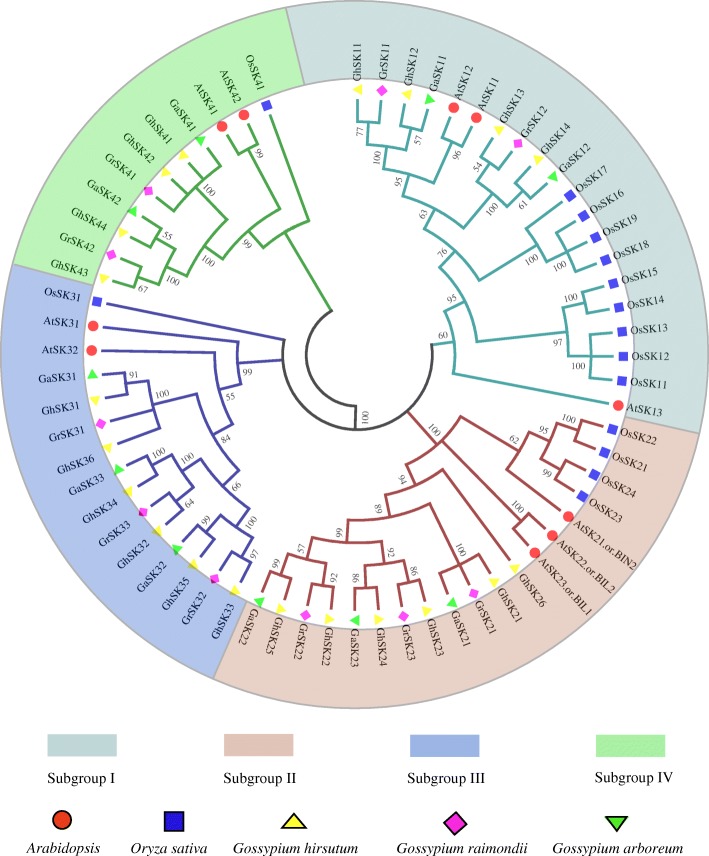
Phylogenetic analysis of GSK3 proteins in *Arabidopsis*, rice, and species of cotton. 10 AtSKs, 15 OsSKs, 20 GhSKs, 10 GaSKs, and 10 GrSKs are divided into four clades. The four clades are respectively colored in cyan, reddish brown, violet, and green

A phylogenetic tree was generated using the neighbor-joining (NJ) **(**Figs. 1 and 3a**)** as well as minimum-evolution (ME) (Additional file 4: Figure S2) method of MEGA6.0. As shown in Figs. 1 and 3a, all sequences were divided into four subgroups (I, II, III, and IV), similar to previous studies [11, 12, 58, 59]. The phylogenetic tree indicated that *GhSK*, *GaSK*, and *GrSK* genes clustered together within each sub-group suggesting a close evolutionary affinity amongst the three cotton species and providing further support for the origins of the tetraploid species from a relatively recent hybridisation between an A-genome progenitor similar to *G. arboreum* and a D-genome progenitor similar to *G. raimondii* [33].

### Chromosomal locations, gene structures, and conserved motifs analysis

**Fig. 2:**
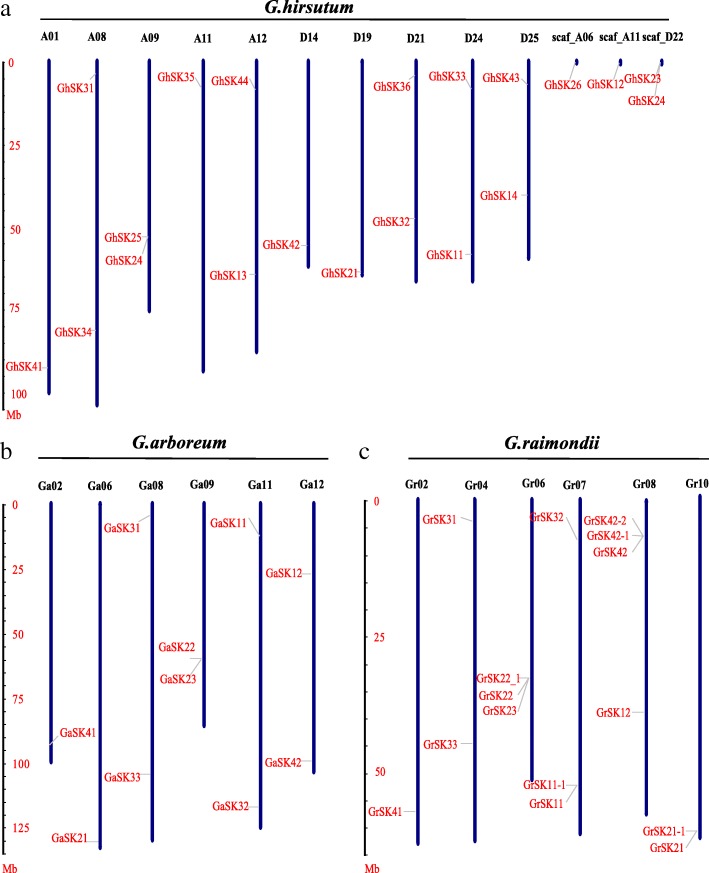
Chromosomal assignment of *GhSKs* (**a**), *GaSKs* (**b**) and *GrSKs* (**c**). The relative chromosomal sizes (unit, Mb) of *G. hirsutum*, *G. arboreum* and *G. raimondii* were calculated from the published genome data of the kinds of cotton

Chromosomal locations of the cotton *GSK* genes using sequencing information for *G. arboreum*, *G. raimondii*, and *G. hirsutum* TM-1 revealed that the genes were unevenly distributed among chromosomes (Fig. 2). Out of the total of 45 cotton *GSK* genes, four *GhSKs* (*GhSK12*, *GhSK23*, *GhSK24*, and *GhSK26*) were assigned to scaffolds not connected to chromosomes (Fig. 2). The other 16 *GhSK* genes were distributed to chromosomes A08, A09 (two genes on each), A12 (two genes), D21 (two genes), D24, D25 (two genes on each), A01, A11, D14 and D19 (Fig. 2a). The *GaSK* genes were localized to chromosomes Ga02, Ga06, and Ga08, Ga11, Ga09 as well as Ga12 (two genes on each) (Fig. 2b), while *GrSK* genes were positioned on chromosomes Gr02 (one gene), Gr04 and Gr10 (two genes on each), Gr06 as well as Gr07 (three genes on each) and Gr08 (four genes) (Fig. 2c).

**Fig. 3:**
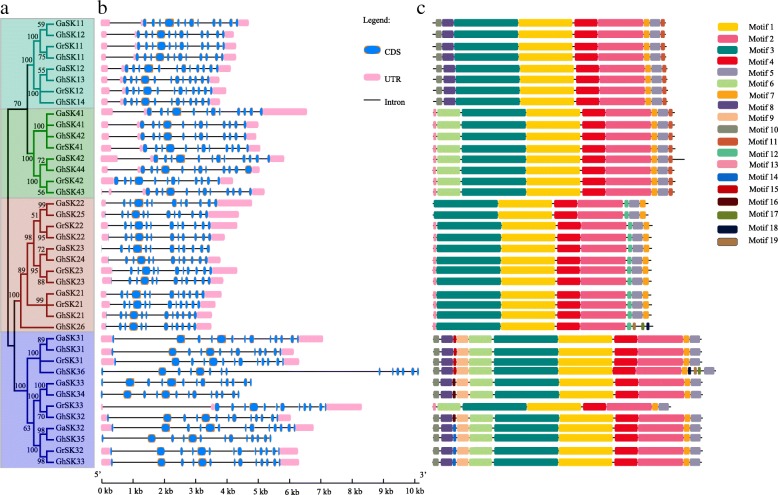
Phylogenetic relationships between the cotton GSKs constructed using the Neighbor-Joining algorithm and Poisson model (**a**), gene structure predictions (**b**) and conversed protein motifs (**c**)

To further clarify the evolutionary relationships (Fig. 3a) among cotton *GSK* genes, genomic information corresponding to 40 (excluding the five different transcripts of *GrSKs*) cotton *GSK* genes extracted from Generic Feature Format (gff3) files were used to examine the structural diversity associated with the *GhSK*, *GaSK*, and *GrSK* genes. The structural analysis (Fig. 3b) indicated that the coding regions of all the cotton *GSK* genes were interrupted by 11–13 introns. The UTR regions of *GhSK34*, *35*, and *36* and *GaSK23* as well as *GaSK33* are undetermined, while other cotton *GSK* genes had UTR region information in the genomic annotation files. In most cases, *GSK* genes within the same sub-family had similar gene structures in regard to the number and length of exons.

Using the MEME online server, 16 conserved domains and protein motifs were observed among 40 cotton GSKs (Fig. 3c), and the respective motif logos are presented in Additional file 5: Figure S3. Members of the same subfamily mostly contained the same motif components, suggesting they might have identical functions. Motifs 1–4 were found in all 40 analyzed GSKs, and constituted the kinase domain of GSK3. Additionally, motif 8 and 10 were present in all proteins of subfamily I and III. Motif 6 was observed in the members of subfamily III and IV. Furthermore, motif 13 was mainly present in subfamily II and IV members (except for GaSK22, GhSK25, and GrSK33), while motif 11 was identified in the proteins of subfamily I and IV. Motif 9 was mainly present in subfamily III members (except for GrSK33). Motif 14, 15, and 16 were present only in some members of subfamily III. The variations in the distribution of motifs indicate that cotton GSKs experienced functional diversification during their evolution.

### Duplicated cotton *GSK* genes and syntenic blocks

During evolution, the two major mechanisms that generate novel genes and contribute to the complexity of the genomes of higher plants are small-scale tandem and large segmental duplications [60]. In this study, 25 pairs of paralogous *GSK* genes resulting from gene duplication events were identified in *G. hirsutum* (Table 2). In contrast, *G. arboreum* and *G. raimondii* consisted of 10 and 22 pairs of paralogs, respectively. Among the 25 pairs of duplicated *GhSK* genes, 14 were observed between different chromosomes, from which 8 pairs were identified between the At and Dt subgenomes. Further, 6 out of the 8 duplicated gene pairs were located between the homologous chromosomes of *G. hirsutum* [34]. *GhSK24*, *GhSK25*, and their paralogs were localized to chromosome A09. Other gene pairs were duplicated either between At to At, or Dt to Dt subgenomes, which might have resulted from the whole genome duplication (WGD) during plant genome evolution.

**Table 2:** Duplicated *GSK* gene pairs identified in *G. hirsutum*, *G. arboreum*, and *G. raimondii*

	Duplicates					
Clade	Gene ID	Gene name	Position	Gene ID	Gene name	Position
II	Ga09G0899	GaSK22	Ga09: 59971361–59,974,663			Ga09: 59971879–59,974,663
II	Ga09G0899	GaSK22	Ga09: 59971361–59,974,663			Ga09: 59989139–59,991,790
II	Ga09G0899	GaSK22	Ga09: 59971361–59,974,663			Ga06: 130211141–130,213,614
II	Ga09G0900	GaSK23	Ga09: 59988594–59,991,790			Ga09: 59989139–59,991,790
II	Ga09G0900	GaSK23	Ga09: 59988594–59,991,790			Ga09: 59971879–59,974,663
II	Ga09G0900	GaSK23	Ga09: 59988594–59,991,790			Ga06: 130211141–130,213,614
IV	Ga02G1328	GaSK41	Ga02:91572951–91,576,357			Ga12: 98906373–98,909,799
II	Ga06G2433	GaSK21	Ga06:130210718–130,213,627			Ga09: 59989139–59,991,777
II	Ga06G2433	GaSK21	Ga06:130210718–130,213,627			Ga09: 59971879–59,974,650
IV	Ga12G2665	GaSK42	Ga12:98904726–98,909,799			Ga02: 91572950–91,576,342
II	Gorai.010G241600.3	GrSK21	Gr10: 61087060–61,090,705			Gr10: 61087549–61,090,443
II	Gorai.010G241600.2	GrSK21–1	Gr10: 61085419–61,090,069			Gr10: 61087549–61,090,014
III	Gorai.004G042400.1	GrSK31	Gr04: 3659148–3,665,477			Gr04: 3659643–3,665,066
III	Gorai.004G156700.2	GrSK33	Gr04: 44445510–44,453,846			Gr04: 44449136–44,452,709
III	Gorai.007G096100.1	GrSK32	Gr07: 7076952–7,083,244			Gr07: 7077235–7,082,655
I	Gorai.007G308500.3	GrSK11–1	Gr07: 52192786–52,196,322			Gr07: 52193148–52,195,963
I	Gorai.007G308500.1	GrSK11	Gr07: 52192786–52,197,103			Gr07: 52193148–52,195,963
IV	Gorai.002G218100.1	GrSK41	Gr02: 56980865–56,985,951			Gr02: 56982189–56,985,586
IV	Gorai.002G218100.1	GrSK41	Gr02: 56980865–56,985,951			Gr08: 6199127–6,202,516
IV	Gorai.008G045800.2	GrSK42–2	Gr08: 6198649–6,203,828			Gr02: 56982189–56,985,579
IV	Gorai.008G045800.2	GrSK42–2	Gr08: 6198649–6,203,828			Gr08: 6199120–6,202,516
IV	Gorai.008G045800.3	GrSK42–1	Gr08: 6198649–6,203,828			Gr02: 56982189–56,985,579
IV	Gorai.008G045800.3	GrSK42–1	Gr08: 6198649–6,203,828			Gr08: 6199120–6,202,516
IV	Gorai.008G045800.6	GrSK42	Gr08: 6198704–6,203,828			Gr02: 56982189–56,985,579
IV	Gorai.008G045800.6	GrSK42	Gr08: 6198704–6,203,828			Gr08: 6199120–6,202,516
I	Gorai.008G136600.1	GrSK12	Gr08: 38585110–38,589,107			Gr08: 38585874–38,588,662
II	Gorai.006G089300.1	GrSK22	Gr06: 32530678–32,535,037			Gr06: 32531407–32,534,186
II	Gorai.006G089300.1	GrSK22	Gr06: 32530678–32,535,037			Gr06: 32547582–32,550,218
II	Gorai.006G089300.2	GrSK22–1	Gr06: 32530521–32,535,037			Gr06: 32531410–32,534,186
II	Gorai.006G089300.2	GrSK22–1	Gr06: 32530521–32,535,037			Gr06: 32547583–32,550,218
II	Gorai.006G089400.1	GrSK23	Gr06: 32546669–32,551,023			Gr06: 32531410–32,534,186
II	Gorai.006G089400.1	GrSK23	Gr06: 32546669–32,551,023			Gr10: 61087562–61,089,975
IV	Gh_A12G0411	GhSK44	A12: 8244999–8,248,430	Gh_D01G1809	GhSK42	D14: 55472542–55,475,929
IV	Gh_A12G0411	GhSK44	A12: 8244999–8,248,430	Gh_A01G1558	GhSK41	A01: 92410496–92,413,866
IV	Gh_A12G0411	GhSK44	A12: 8244999–8,248,430	Gh_D12G0407	GhSK43	D25: 6533936–6,537,332
II	Gh_D06G2142	GhSK21	D19: 63261021–63,263,915	Gh_A06G2020	GhSK26	Scaf1340_A06: 43051–45,948
I	Gh_D11G2830	GhSK11	D24: 58071261–58,074,075	Gh_A11G3270	GhSK12	Scaf3045_A11: 160891–163,705
III	Gh_A08G0285	GhSK31	A08: 3329052–3,334,479	Gh_D08G0378	GhSK36	D21: 3871235–3,880,133
IV	Gh_D12G0407	GhSK43	D25: 6533937–6,537,332	Gh_D01G1809	GhSK42	D14: 55472542–55,475,929
IV	Gh_D12G0407	GhSK43	D25: 6533937–6,537,332	Gh_A01G1558	GhSK41	A01: 92410496–92,413,866
IV	Gh_D12G0407	GhSK43	D25: 6533937–6,537,332	Gh_A12G0411	GhSK44	A12: 8244998–8,248,430
III	Gh_D08G0378	GhSK36	D21: 3871236–3,880,133	Gh_A08G0285	GhSK31	A08: 3329051–3,334,479
I	Gh_D12G1230	GhSK14	D25: 40170076–40,172,846	Gh_A12G1106	GhSK13	A12: 64114145–64,116,909
IV	Gh_D01G1809	GhSK42	D14: 55472543–55,475,936	Gh_A01G1558	GhSK41	A01: 92410496–92,413,873
IV	Gh_D01G1809	GhSK42	D14: 55472543–55,475,936	Gh_D12G0407	GhSK43	D25: 6533943–6,537,332
II	Gh_A09G0712	GhSK25	A09: 52841598–52,844,894			A09: 52842113–52,844,894
II	Gh_A09G0712	GhSK25	A09: 52841598–52,844,894	Gh_A09G0713	GhSK24	A09: 52861923–52,864,569
II	Gh_A09G0712	GhSK25	A09: 52841598–52,844,894	Gh_D09G2469	GhSK22	Scaf4332_D22: 137948–140,717
II	Gh_A09G0712	GhSK25	A09: 52841598–52,844,894	Gh_D09G2468	GhSK23	Scaf4332_D22: 123123–125,754
II	Gh_A09G0713	GhSK24	A09: 52861382–52,864,569			A09: 52842113–52,844,894
II	Gh_A09G0713	GhSK24	A09: 52861382–52,864,569			A09: 52861923–52,864,569
II	Gh_A09G0713	GhSK24	A09: 52861382–52,864,569	Gh_D09G2469	GhSK22	Scaf4332_D22: 137948–140,717
II	Gh_A09G0713	GhSK24	A09: 52861382–52,864,569	Gh_D09G2468	GhSK23	Scaf4332_D22: 123123–125,754
III	Gh_D11G0907	GhSK33	D24: 7839230–7,844,650	Gh_A11G0778	GhSK35	A11: 7649122–7,654,545
III	Gh_A08G1158	GhSK34	A08: 81021333–81,025,764	Gh_D08G1440	GhSK32	D21: 47361119–47,366,590
IV	Gh_A01G1558	GhSK41	A01: 92410497–92,413,873	Gh_D01G1809	GhSK42	D14: 55472542–55,475,936
IV	Gh_A01G1558	GhSK41	A01: 92410497–92,413,873	Gh_D12G0407	GhSK43	D25: 6533943–6,537,332
III	Gh_A11G0778	GhSK35	A11: 7649123–7,654,545	Gh_D11G0907	GhSK33	D24: 7839229–7,844,650
III	Gh_D08G1440	GhSK32	D21: 47361120–47,366,590	Gh_A08G1158	GhSK34	A08: 81021332–81,025,764
I	Gh_A12G1106	GhSK13	A12: 64114146–64,116,909	Gh_D12G1230	GhSK14	D25: 40170075–40,172,846
II	Gh_A06G2020	GhSK26	Scaf1340_A06: 43052–45,877	Gh_D06G2142	GhSK21	D19: 63261091–63,263,915
I	Gh_A11G3270	GhSK12	Scaf3045_A11: 160892–163,705	Gh_D11G2830	GhSK11	D24: 58071260–58,074,075
II	Gh_D09G2468	GhSK23	Scaf4332_D22: 123124–126,295			A09: 52861923–52,864,569
II	Gh_D09G2468	GhSK23	Scaf4332_D22: 123124–126,295			A09: 52842113–52,844,894
II	Gh_D09G2468	GhSK23	Scaf4332_D22: 123124–126,295	Gh_D09G2469	GhSK22	Scaf4332_D22: 137948–140,717
II	Gh_D09G2469	GhSK22	Scaf4332_D22: 137949–141,233			A09: 52861923–52,864,569
II	Gh_D09G2469	GhSK22	Scaf4332_D22: 137949–141,233			A09: 52842113–52,844,894
II	Gh_D09G2469	GhSK22	Scaf4332_D22: 137949–141,233	Gh_D09G2468	GhSK23	Scaf4332_D22: 123123–125,754

**Fig. 4:**
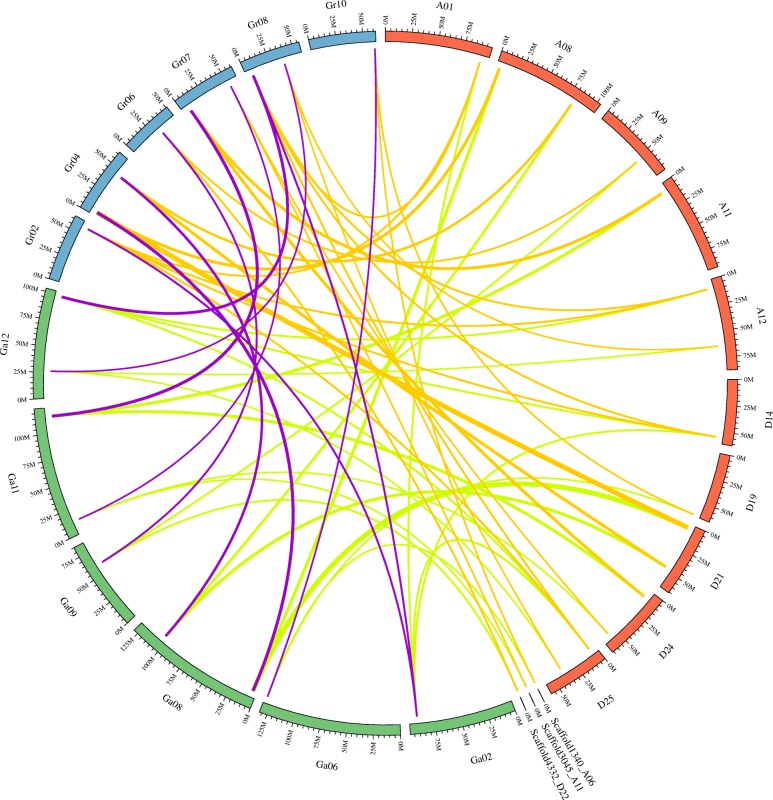
Orthologous *GSK* genes identified in *G. hirsutum*, *G. arboreum* and *G. raimondii*. Links between *G. hirsutum* and *G. arboreum* are colored in cyan, *G. hirsutum* and *G. raimondii* in orange, *G. arboreum* and *G. raimondii* in light purple. Chromosomes of *G. hirsutum*, *arboreum* and *raimondii* were respectively colored in orange-red, green and blue

Syntenic blocks consist of conserved genes arranged similarly in chromosomes of different species. In this study, 26, 28 and 13 conserved non-nested syntenic blocks around GSK genes were predicted between the genomes of *G.hirsutum* and *G. arboreum*, *G. hirsutum* and *G. raimondii*, and *G. arboreum* and *G. raimondii*, respectively (Fig. 4). The detailed information regarding each syntenic block is provided in Additional file 6: Table S3. The *G. hirsutum* genes on chromosomes A01, A08, A09, A11, A12, D14, D19, D21, D24, and D25 as well as scaffold 1340_A06, 3045_A11, and 4332_D22 were likely derived from conserved blocks on *G. arboreum* chromosome Ga02, Ga06, Ga08, Ga09, Ga11, and Ga12. Meanwhile, genes on *G. raimondii* chromosome Gr02, Gr04, Gr06, Gr07, Gr08, and Gr10 were predicted to possess ortholog genes on *G. hirsutum* chromosomes as well as orthologs of *GaSKs*.

### *GhSK* expression profiles in various tissues and organs

**Fig. 5:**
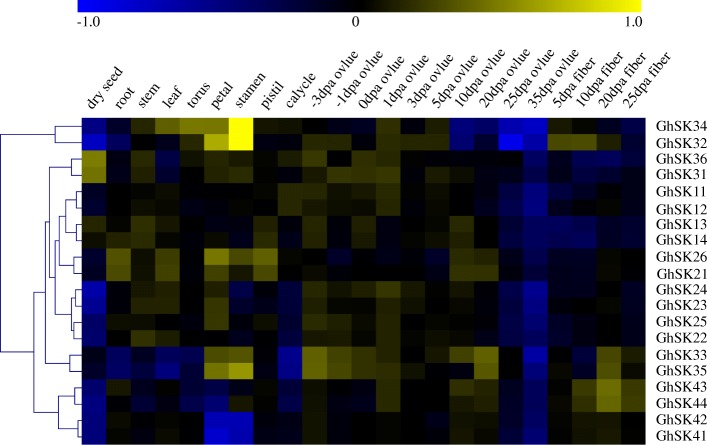
Hierarchical clustering of gene expression profiles of all *GhSK* members in 23 diverse organs/tissues (dry seed, root, stem, leaf, torus (receptacle), petal, stamen, pistil, calycle (epicalyx), 10 stages of ovule and 4 stages of fiber). The FPKM values, log10-transformed, were utilized to generate the heat map. The blue colors indicate low expression levels, while yellow represents high expression levels

Different tissue- and organ-specific gene expression patterns might be an indicator of distinct biological functions. Heat maps of transcriptomic data from the NCBI database revealed that most of the genes assigned to the same subfamily were clustered together and had similar expression profiles (Fig. 5). For example, most of *GhSK* genes were expressed at relatively low levels in 25, 35 dpa ovules and fiber tissues. The exception being subfamily IV members, as well as *GhSK33* and *GhSK35* in 10 and 20 dpa ovule, *GhSK34* in early stage fiber and *GhSK32* in 5, 10, and 20 dpa fiber tissues where their expression was often significant. Most of *GhSK* genes had relatively high expression levels in − 1~ 20 dpa ovules. Additionally, *GhSK32*, *GhSK33*, *GhSK34*, and *GhSK35* were highly expressed in petal and stamen tissues. Apart from subfamily IV members, other *GhSKs* had relatively high, but uneven expression patterns in various floral organs. Most of the *GhSK* members showed poor expressions in the dry seed, while some exhibited slightly higher expression in the calycle (epicalyx). Nearly all the *GhSKs* had relatively high expression levels in leaf and stem, excluding *GhSK31*, *GhSK33*, *GhSK35*, *GhSK36* and members of subfamily IV. Furthermore, only *GhSK14*, *GhSK21*, *GhSK25*, *GhSK42*, *GhSK43* exhibited relatively high expression levels in root tissue.

**Fig. 6:**
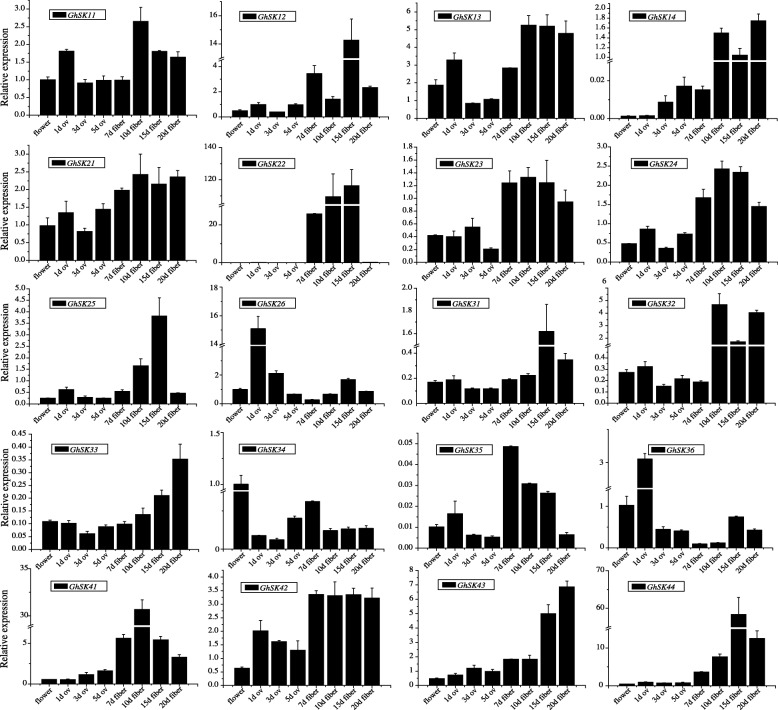
Validation by qRT-PCR of tissues/organ-specific expression patterns of *GhSK* genes (flower, ovules of 1, 3, and 5 dpa, fibers of 7, 10, 15 and 20 dpa samples of CCRI24 were used). Data are the mean ± SE of three independent experiments

The different members of the same gene family can exhibit diverse expression patterns in various tissues/organs and play different physiological functions [61]. To further validate the transcriptomic data, the expression levels of 20 *GhSK* genes were analyzed in 10 different tissues/organs, including flower, ovule (1, 3, and 5 dpa), and fiber (7, 10, 15, and 20 dpa) by Q-PCR (Fig. 6). Here, 13 of the 20 genes (*GhSK12*, *13*, *14*, *22*, *23*, *24*, *25*, *32*, *35*, *41*, *42*, *43*, and *44*) were preferentially or relatively highly expressed in different fiber development stages. The *GSK* gene expression was lower in whole flower tissues than in the isolated growing fibers.

### Analysis of *GSK* promoter regions

Abundant hormone- and stress-related *cis*-elements, including *cis*-elements that confer high transcript levels and MYB-binding sites were detected in the regions 2 kb upstream of the transcription start site of different GhSK genes (Additional file 7: Figure S4 and Additional file 8: Table S4). The phylogenetically similar genes shared identical *cis*-elements. All of the *GhSK* promoter regions contained more than one *cis*-element that confers high transcript level. Additionally, heat-, defense-, stress-, salicylic acid-, and auxin-responsive *cis*-elements were identified in the promoter regions of almost all *GhSK* genes, except for *GhSK44* (no heat-responsive element), *GhSK13* and *32* (no defense- and stress-responsive elements), *GhSK13*, *14* and *43* (no salicylic acid-responsive element), and *GhSK11, 12, 43,* and *44* (no auxin-responsive element). Furthermore, wound-responsive elements were detected only in the promoter regions of *GhSK21, GhSK26*, and *GhSK32*, while only *GhSK23 and 33* contained an ABA-responsive element. Moreover, 9 and 13 out of 20 *GhSK* genes contained low temperature- and GA-responsive elements respectively, while methyl jasmonate- and fungal elicitor-responsive *cis*-element were observed in 8 and 9 genes respectively. MYB-binding site or MYB binding site involved in regulating flavonoid gene expression was observed in 4 *GhSKs* promoter regions, while elements of drought-inducible MYB binding site were observed in 13 *GhSKs* promoter regions. Additionally, the ethylene-responsive element was identified in 8 *GhSKs* promoter regions. Furthermore, three kinds of tissue-specific (shoot, meristem, and seed) elements were found in the promoter regions of some of the *GhSK* genes.

### *GSK* gene expression patterns in response to several abiotic stresses

**Fig. 7:**
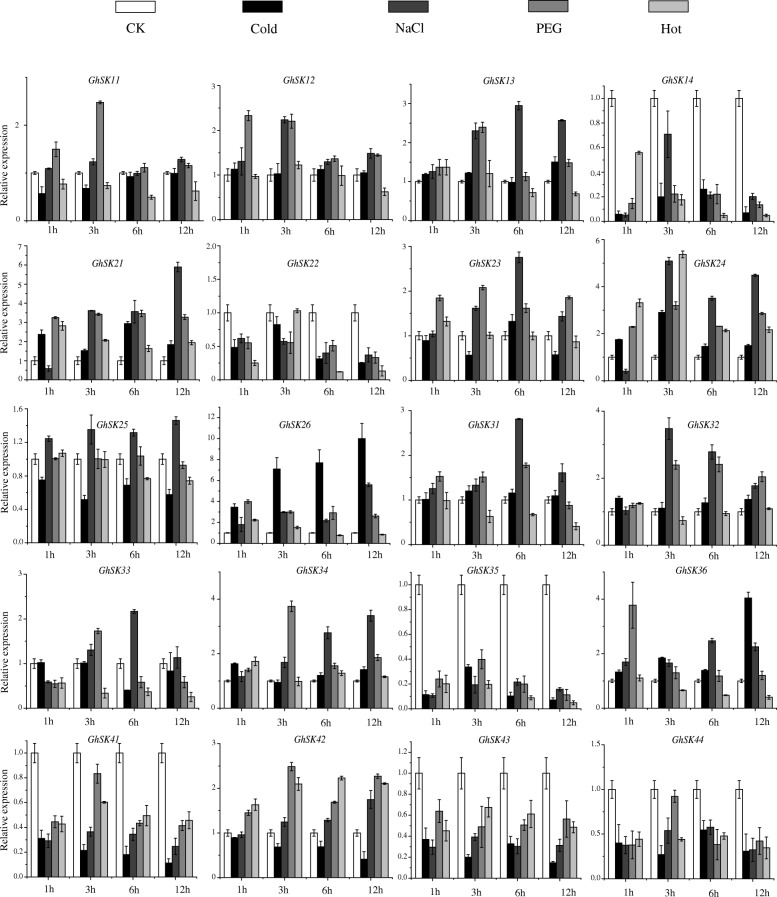
20 *GhSKs* relative expression patterns in response to cold, NaCl, PEG and heat stresses were analyzed by qRT-PCR. The relative expression levels of CK (0 h) were normalized to 1. Data were the mean ± SE of three independent experiments

To explore the physiological and functional relevance of *GhSK* genes, we investigated the expression patterns of 20 *GhSK* genes under different environmental stresses [i.e., Cold (4 °C), salinity, simulated drought (PEG), and heat (38 °C)] (Fig. 7). Among the 20 genes, expression levels of six *GhSK* (i.e., *GhSK14*, *22*, *35*, *41*, *43*, and *44*) genes were down-regulated by all imposed stresses. Not all *GhSK* genes were induced or repressed by temperature stresses. Among them, *GhSK21*, *24*, *26*, and *36* were cold induced, *GhSK21*, *24* and *42* were up-regulated by heat stress, while the expression levels of *GhSK31*, *33*, and *36* were repressed under heat condition. Except for *GhSK25*, *31*, and *33*, the expression levels of the other 11 *GhSK* genes (excluded those six uniformly down-regulated genes mentioned above) were up-regulated on exposure to PEG. *GhSK11*, *12*, and *13* were slightly induced by PEG treatment during the first 3 h. *GhSK21*, *GhSK24*, and *GhSK26* were highly responsive to PEG treatments, with 3-fold, 2-fold, and 4-fold increases, respectively, irrespective of different times after treatment. Unlike *GhSK31* and *33*, expression levels of the other *GhSK3s* exhibited more than 2-fold increases at 3 h after PEG treatment excluding *GhSK36* that peaked at 1 h with about 4-fold changes. The expression level of *GhSK42* was only slightly affected by PEG at 1 h but was subsequently induced, reaching its peak level (approximately 2.5-fold increase) at 3 h. For salinity stress, expression levels of *GhSK11*, *25*, and *42* were not significantly induced at any time point. However, *GhSK12*, *24*, and *32* expression levels increased during the first 3 h and then decreased slightly. Additionally, the expression levels of *GhSK13*, *23*, *31*, *33* and *36* were up-regulated over the first 6 h (approximately 3-fold, 3-fold, 3-fold, 2.2-fold, and 2.3-fold increases, respectively) and then decreased. Meanwhile, *GhSK21*, *26*, *34* as well as *42* expression levels were up-regulated gradually and finally peaked at 12 h after salinity treatment.

### Regulatory sub-networks involving *GhSK* and other *G. hirsutum* genes

**Fig. 8:**
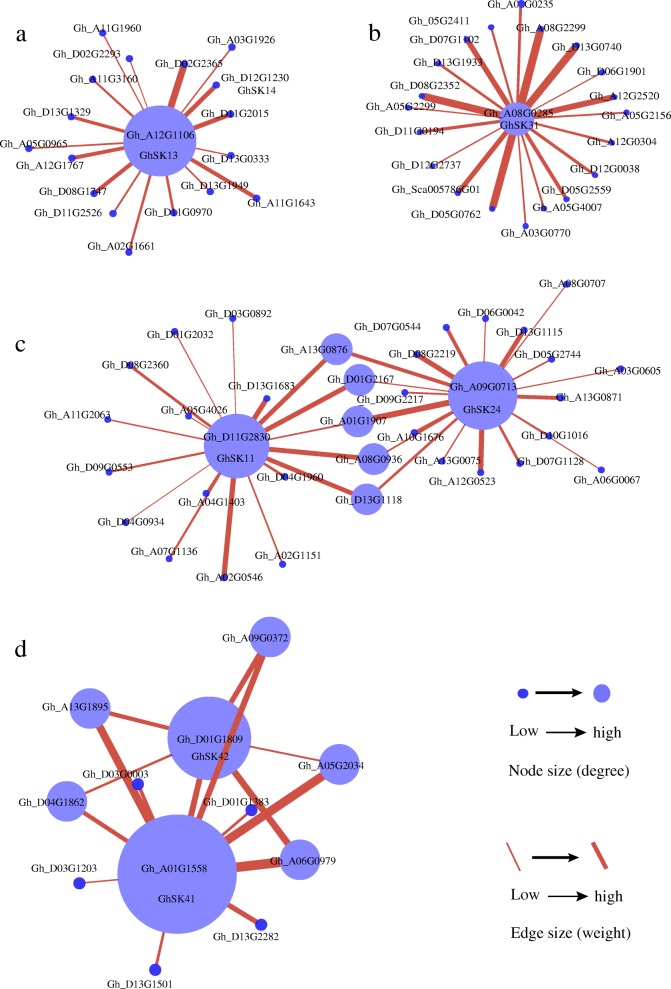
The weighted gene co-expression analysis modules of *GhSK* genes visualized by Cytoscape software. **a** Co-expression sub-network for *GhSK13*. **b** Co-expression sub-network for *GhSK31*. **c** Co-expression sub-network for *GhSK14* and *GhSK24*. **d** Co-expression sub-network for *GhSK42* and *GhSK41*. Nodes are genes with node size related to the number of connections of that gene to other co-expressed genes. Nodes are connected by lines (edges) with the weight of the line determined by the size of the correlation coefficient with thicker lines having a higher correlation and hence higher certainty

Co-expressed genes exhibit similar increases or decreases in transcription levels in different samples [62, 63]. The *GSKs* are known to influence several developmental, signaling, and physiological processes in plants [2]. The co-expression between *GhSK* and other *G. hirsutum* genes were analyzed using the R package (WGCNA), and the co-expressed genes linking to other *GhSK* genes (*GhSKs* linkers) were identified and annotated (Fig 8**,** Additional file 9: Figure S5a, S5b, Additional file 10: Figure S6 and Additional file 11: Table S5.1, S5.2 and S5.3). We detected five modules (blue, brown, green, turquoise and yellow), in which nine *GhSK* genes were co-expressed with other *G. hirsutum* genes. Surprisingly, the *GhSK32* (found in brown module) was co-expressed with 2781 *G. hirsutum* genes across all the analyzed samples (Additional file 10: Figure S6 and Additional file 11: Table S5.1, S5.2, and S5.3). The *GhSK34* (also present in the brown module) had 2142 co-expressed genes (Additional file 10: Figure S6 and Additional file 11: Table S5.3). These genes in the brown module were enriched in 55 GO terms (*FDR < 0.05*) (i.e., 30, 20, and 5 related to molecular functions, biological processes, and cellular components, respectively). Additionally, *GhSK13* was co-expressed with 17 genes (including *GhSK14*) (Fig. 8a), similarly, *GhSK31* was co-expressed with 20 genes (Fig. 8b). *GhSK11*, *GhSK25*, and *GhSK24* were co-expressed with 19, 1 (Gh_A04G1034), and 20 other *G. hirsutum* genes, respectively (Fig. 8c), meanwhile, *GhSK42* and *GhSK41* were co-expressed with 6 and 11 genes, respectively (Fig. 8d). Among these genes, *GhSK11* and *GhSK24* were indirectly co-expressed with each other, as were *GhSK42* and *GhSK4* (directly/indirectly). Similarly, *GhSK32* co-expressed with *GhSK34* both directly and indirectly (Additional file 10: Figure S6 and Additional file 11: Table S5.3). The co-expressed genes in the brown module were mainly enriched (Top20 GO) for protein phosphorylation, protein kinase activity, integral component of membrane, Calcium ion binding, transmembrane transport, and enzyme inhibitor activity (Additional file 9: Figure S5a). Further, these were also significantly enriched in pentose and glucoronate interconversion, plant-pathogen interaction, as well as starch and sucrose metabolism pathways (Top20 KEGG) (Additional file 9: Figure S5b). The GO (*FDR < 0.05*) and KEGG (*p < 0.05*) enrichment results for the other target genes are presented in Additional file 11: Table S5.1 and S5.2.

## Discussion

Protein kinases form a large family of enzymes that mediate eukaryotic cell responses to external stimuli [64]. To date, several protein kinase family members have been identified, including GSK3, MAPKKK, Pto-like protein kinase, and PP2C [65–68]. Among these enzymes, GSK3 reportedly regulates different physiological and developmental processes in mammals and plants [2, 9, 45, 69, 70]. However, the genome-wide characterization of GSK3-like kinases has been limited to model plants, even though these enzymes were first reported decades ago [67].

### The cotton GSK gene family expanded but remained highly conserved during evolution

The availability of high-quality whole draft genome sequences has enabled the characterization of *G. aboreum*, *G. raimondii*, and *G. hirsutum* genes. Interestingly, the number of *G. hirsutum GSK* genes almost equals to the sum of *G. arboreum* and *G. raimondii* genes, possibly because of its formation as an allotetraploid following hybridization of A and D genome progenitors [33, 71]. Consistent with the names of corresponding *A. thaliana* and rice genes [45], we renamed the cotton *GSK* genes as *GhSKs* (i.e., *G. hirsutum Shaggy/GSK3-like kinases*), *GaSKs* and *GrSKs* (Table 1). Additionally, the cotton *GSK* genes were divided into four subfamilies in accordance with the *A. thaliana* and rice *GSK* genes. We observed that subfamily II was the largest containing 14 members, then subfamily III with 12 members, IV and I comprised of 10 and 9 members respectively (Table 1 and Fig. 1). Moreover, the sub-cellular location of almost all GSKs was predicted to be in the nucleus, membrane and cytoplasm, except for GaSK23 (cytoplasmic) (Table 1 and Fig. 1). Additionally, the kinase domains of AtSKs and OsSKs were 65–72% similar to human GSK3β [45]. Here, we aligned the peptide sequences of AtSKs, OsSKs, GaSKs, GrSKs, and GhSKs. All of these GSKs contain related conserved domains and residues, suggesting these enzymes are highly conserved across species and that of the GSK family existed before the separation of these different species [2].

Consistent with the AtSKs, cotton GSKs are clustered into four clades [45]. The results of phylogenetic analysis and the presence of conversed domains suggested that the GSKs may have analogous biological functions [2]. However, various bootstrap values in the phylogenetic tree were low, potentially because of a relatively low similarity in the protein sequences of different species. This implied there was considerable variability in the *GSK* gene sequences, even though the protein motifs were highly conserved (Fig. 1). Furthermore, the cotton *GSK* genes clustered in the same subfamily were observed to encode proteins with similar motifs and protein architectures, suggesting the protein structures were conserved among members of the same subfamily. Nevertheless, there was significant diversity in the N-terminal domains of GSKs between different subfamilies, which was consistent with the findings of Forde and Dale [72]. Protein kinases are important for regulating protein phosphorylation in many cellular activities [73]. Integrating the results of conserved protein motifs, multiple sequence alignment, and previously published research [2], we speculate that a functional GSK kinase domain (motifs 1 to 4) was present in all cotton GSKs although we have not confirmed this enzymatically (Fig. 3c and Additional file 5**:** Figure S3). Additionally, most of these GSKs also contain serine and/or threonine kinase active sites and tyrosine residues, which could be phosphorylated by other kinases to modulate their enzymatic activity. This is believed to occur in all GSK3s and is required for the full kinase activity of human GSK3β. [74, 75].

### Gene duplicates and synteny blocks were detected between cotton *GSK* genes

Allotetraploid cotton was generated from the fusion of two diploid cotton species, with chromosomes 1–13 and 14–26 derived from A and D genomes, respectively [33, 71]. In our study, *GSK* genes were unevenly distributed on the chromosomes of the three analyzed cotton species (diploid *G. arboreum* and *G. raimondii* as well as allotetraploid *G. hirsutum*). Thus, *GSK* gene duplication events might have occurred in these chromosomes during evolution, with neo- or sub-functionalization of these genes.

Orthologs are genes in different genomes that were derived from the same ancestral gene; they encode proteins with similar biological functions, whereas paralogs are defined as genes derived from a single gene via a duplication event, encoding proteins with different functions [76, 77]. During evolutionary processes, new genes were generated as a result of large segmental and small-scale tandem duplications or polyploidization [60, 78]. Duplicated genes are often found to be involved in the formation of paralogous genes present in gene families [78]. Tandem duplication usually results from unequal crossovers [79] and multiple occurrences of these can result in the expansion or reduction of different gene family members [60]. Here, *G. arboreum*, *G. ramondii*, and *G. hirsutum* contain more than one *GSK* gene clusters and genes within them with similar intron numbers suggesting that these duplicated genes might be the result of unequal crossovers. Additionally, recent studies demonstrated that the *G. arboreum* and *G. raimondii* genomes underwent at least two whole-genome duplication events [35, 36]. Although the total number of *G. hirsutum* genes dramatically expanded after polyploidization and duplication, gene loss also occurred during the evolution of upland cotton [39]. These might be the cause of the greater number of paralog gene pairs (25) in upland cotton compared to *G. arboreum* (10) and the uneven distribution of *GSK* genes on different chromosomes. Considering that orthologs might exhibit the same biological functions over the course of evolution [80], the syntenic blocks between *G. hirsutum* and *G. arboreum*, *G. hirsutum* and *G. raimondii*, and *G. arboreum* and *G.raimondii* were identified. All *GhSK* genes had at least one syntenic gene in *G. arboreum* and *G. raimondii*. Further, most of the highly similar gene pairs in regions around GSK genes found by comparing *G. arboreum* and *G. hirsutum* (red), *G. raimondii* and *G. hirsutum* (blue), and *G. arboreum* and *G. raimondii* (orange) in our study coincided with previous synteny analyses of various published cotton genomes [19, 34, 43], marked in different colors mentioned above and detailed in Additional file 6: Table. S3, suggesting that most of the cotton *GSK* loci were conserved during evolution.

### *GhSKs* are involved in fiber development and abiotic stresses response

Variations in the expression patterns of *GSK* family members in various tissues/organs may be related to their functional differences [61]. To further characterize the potential regulatory functions of *GhSKs* affecting cotton growth and development, the expression patterns of 20 *GhSK* genes were analyzed using both transcriptomic data and qRT-PCR data. Similar tissue- and organ-specific expression patterns were observed for the same *GhSK* gene based on either transcriptome or qRT-PCR data. Furthermore, *GhSK22*, *41*, and *GhSK44* were preferentially expressed in fibers. The *GhSK12*, *25*, and *32* expression levels were also relatively high in different fiber tissues. *GSK* genes were previously reported to be involved in BR signaling in flowering plants [2]. These kinases may help to control cell elongation in plants because BRs regulate cell elongation [81]. However, gene expression levels of most *GhSKs* were not induced or repressed by exogenous BL treatment (Additional file 12: Figure S7). Previous reports indicated that brassinolide treatment reduced in vivo phosphorylation of BIN2, a special GSK3 member, and inhibited its function [82]. Similarly, BL treatment has been reported to lead to reductions in the protein accumulation and to inhibit the phosphorylation of AtSK12 [82]. The BR signal transduction pathway starts from a receptor kinase (BRI) to BR responsive transcription factors BZR1 and BES1, through a process of phosphorylation and dephosphorylation [83–85]. Last but not the least, we checked the differential expression levels of *AtSKs* in previously published transcriptome data of BL treated *Arabidopsis* [86], but similarly saw no evidence of transcriptional induction or repression of *AtSKs*. We speculate that BRs might affect GSKs at the protein and post-translational modification levels, rather than at the level of transcriptional regulation. Additionally, BIN2 functions in the crosstalk between BRs and auxin. *AtSK21/BIN2* expression accelerates lateral root formation via the activation of ARF7 and ARF19, which are two factors involved in auxin-mediated lateral root development [87]. Meanwhile, TGA elements described as auxin-responsive elements were found in almost all *GhSK* gene promoter regions, and previous research has demonstrated that NAA (a synthetic auxin) enhances fiber elongation by suppressing secondary wall cellulose synthesis [88]. Furthermore, the regulation of auxin biosynthesis in ovule epidermal cells of cotton can enhance fiber quality and yield [89]. Additionally, ethylene was reported as playing a major role during fiber elongation [90], and ERE (ethylene responsive elements) were found in 8 *GhSK* promoter regions. The current evidence suggests that *GhSKs* may therefore mediate fiber development and elongation.

Plant responses to abiotic and biotic stresses are found to be regulated by GSK3 [2], although not in a consistent fashion [12, 15, 91–95]. Previous studies confirmed that *GSK3* expression is up-regulated by salt stress in *A. thaliana* [12], rice [91], wheat [96], and sugarcane [95]. Moreover, the expressions of *AtSK13*, *31*, and *42* are also up-regulated by osmotic stress, while *OsGSK3* expression is down-regulated under drought conditions [91]. Consistent with these findings, we determined that the transcription of most *GhSK* genes were up-regulated in response to several abiotic stresses. The opposite trend was observed for six other *GhSK* genes. In contrast, *GhSK25* expression was rarely affected under abiotic stress conditions (Fig. 7). Another study indicated that *OsGSK3* expression is up-regulated on exposures to salt, cold, and drought stresses, mechanical injury, and exogenous ABA [6]. These results imply the encoded kinase activates signal cascade during stress. Besides, over-expressing *TaSK5* cDNA in *A. thaliana* enhances the resistance of the transgenic plants to salinity and drought stresses, while their tolerance to freezing stress is retained [97]. Coupled with these results, we may conclude that stress-responsive *cis*-elements in the *GhSK* promoter regions play crucial roles during plant responses to various abiotic stresses.

### Several fiber-related genes are co-expressed with *GhSKs*

Glycogen synthase kinase 3 is a serine/threonine kinase with several vital roles in animals [93]. There are about 18 proteins verified as direct substrates of GSK3. This kinase is associated with multiple molecular and cellular mechanisms affecting glycogen metabolism, cell cytoskeleton stability, as well as the regulation of cell division, differentiation, and apoptosis [98]. In the current study, a coexpressed network analyzed by WGCNA indicated that 9 *GhSK* genes were co-expressed with one or more other *G. hirsutum* genes. Additionally, a KEGG enrichment analysis (*p < 0.05*) revealed that these 9 *GhSK* genes could be involved in stress responses, phytohormone signal transductions, and/or the Wnt signaling pathway. Moreover, the genes co-expressed in the brown module containing *GhSK32* and *GhSK34* were significantly enriched for protein phosphorylation (GO:0006468), protein kinase activity (GO:0004672), integral component of membrane (GO:0016021), Calcium ion binding (GO:0005509), transmembrane transport (GO:0055085) and enzyme inhibitor activity (GO:0004857). The genes co-expressed with *GhSK41* and *GhSK42* were enriched for functions related to cytoskeleton organization (GO:0007010) and microtubule binding (GO:0008017). Cortical microtubules and newly deposited cellulose microfibrils are transversely oriented relative to the growth axis of fiber cell, and are critical for longitudinal expansion [99]. In addition, the temporal and spatial Ca^2+^ concentration changes in the fiber tip are converted through calcium sensors, such as calmodulins, calcium-dependent protein kinases, and calcineurin B-like proteins that have been shown to be necessary for maintaining fast cell elongation [23]. Furthermore, ethylene has also been shown to promote fiber elongation by activating genes involved in cytoskeleton organization and cell wall loosening [23, 90]. These findings may help to clarify the potential functions of GhSKs in fiber development and will lay the foundations for further molecular and functional analysis of *GSK* genes in cotton.

## Conclusions

Previous research has shown that *GSK* genes have significant functions in regulating plant growth and development, as well as responses to various abiotic stresses. In the present study, *GSK* genes were shown to be highly conserved among *Arabidopsis*, rice and three cotton species (*G. hirsutum*, *G. raimondii*, and *G. arboreum*). Whole genome duplication was speculated as the major impetus for the expansion of the *GSK* gene family in cotton. Additionally, the duplicated *GhSK* genes might have experienced functional diversification as the duplicated gene pairs exhibited disparate expression patterns in different tissues and organs. Further, some *GSK* gene members were shown to be involved in fiber development as well as stress responses.

## Additional files

Additional file 1: **Table S1.1** Gene accession numbers and sample information of transcriptome data used in our research. **Table S1.2** FPKM values of transcriptome data used in our research. (XLSX 16765 kb)

Additional file 2: **Table S2.** FPKM values of different tissues obtained from published transcriptome data. (XLSX 16 kb)

Additional file 3: **Figure S1.** Multiple sequence alignments of the kinase domains of Arabidopsis, rice, and cotton. Amino acids are shaded in black, dark and light grey based on sequence similarity (high to low). The kinase-inactivating mutation *bin2* that suppresses gain of function are highlighted in light green. The conserved phosphate-binding residues are highlighted in orange. The conversed tyrosine phosphorylated residue is highlighted in red. The conserved TRRE box in which the three residues (T, E, and E) that lead to BIN2 gain of function are included are highlighted in green. (PDF 3258 kb)

Additional file 4: **Figure S2.** Phylogenetic analysis of GSK3 proteins in *Arabidopsis*, rice, and species of cotton. 10 AtSKs, 15 OsSKs, 20 GhSKs, 10 GaSKs, and 10 GrSKs are divided into four clades. The four clades are respectively colored in cyan, reddish brown, violet, and green. The tree was constructed by Minimum-Evolution using Poisson model of MEGA 6.0. (PDF 301 kb)

Additional file 5: **Figure S3.** Motif logos of 16 conserved motifs found in cotton GSK proteins. (PDF 817 kb)

Additional file 6: **Table S3.** Orthologous *GSK* genes identified in *G. hirsutum*, *G. arboreum* and *G. raimondii*. Orthologous genes were highlighted on condition that they were coincident with the results analyzed by MCScanX software: orthologous gene pairs between *G. hirsutum* and *G. arboreum* were highlighted in red, *G. hirsutum* and *G. raimondii* in blue, as well as *G. arboreum* and *G. raimondii* in orange. (XLSX 18 kb)

Additional file 7: **Figure S4.** Cis-regulatory elements predicted in the promoter regions of cotton *GSK* genes. (PDF 173 kb)

Additional file 8: **Table S4.** Cis-regulatory elements found in the promoter regions of cotton *GSK* genes. (XLSX 33 kb)

Additional file 9: **Figure S5.** The GO (a) and KEGG (b) enrichment of weighted co-expressed genes of the brown module *(GhSK32)* as predicted by “WGCNA” R package with published transcriptomics data. (PDF 149 kb)

Additional file 10: **Figure S6.** The weighted gene co-expression analysis sub-network of *GhSK32* and *GhSK34* were visualized by Cytoscape software. (PDF 7454 kb)

Additional file 11: **Table S5.1** GO annotations of the co-expressed genes involved in the weighted gene co-expression sub-network of *GhSKs*. **Table S5.2** KEGG annotations of the co-expressed genes involved in the weighted gene co-expression sub-network of *GhSKs*. **Table S5.3** Linkers co-expressed with *GhSK32* and *GhSK34. (XLSX 201 kb)*

Additional file 12: **Figure S7.** 20 *GhSKs* expression patterns in response to BL treatment were analyzed by qRT-PCR. The relative expression levels of CK (0 h) were normalized to 1. Data are the mean ± SE of three independent experiments. (PDF 459 kb)

## Abbreviations

*A. thaliana*: *Arabidopsis thaliana*
ABA: Abscisic acid
BIN2: Brassinosteroid-Insensitive 2
BL: Brassinolide
BLAT: BLAST-Like Alignment Tool
BR: Brassinosteroid
Dpa: Day post-anthesis
G6PD: Glc-6-phosphate dehydrogenase
GA: Gibberellins
GaSKs: *Gossypium arboreum* Shaggy/GSK3-like kinases
GhSKs: *Gossypium hirsutum* Shaggy/GSK3-like kinases
GO: Gene Ontology
GrSKs: *Gossypium raimondii* Shaggy/GSK3-like kinases
GSK3: Glycogen synthase 3/shaggy-like kinase
KEGG: Kyoto Encyclopedia of Genes and Genomes
PEG: Polyethylene glycol
qRT-PCR: Quantitative real-time polymerase chain reaction
WGCNA: Weighted gene co-expression network analysis
