# Supplementary figures and images for: Genome-wide characterization and phylogenetic analysis of GSK gene family in three species of cotton: evidence for a role of some GSKs in fiber development and responses to stress
Source: BMC Plant Biol. 2018 Dec 4;18:330. doi: 10.1186/s12870-018-1526-8 (PMC6280398; doi:10.1186/s12870-018-1526-8)

Fig. S2

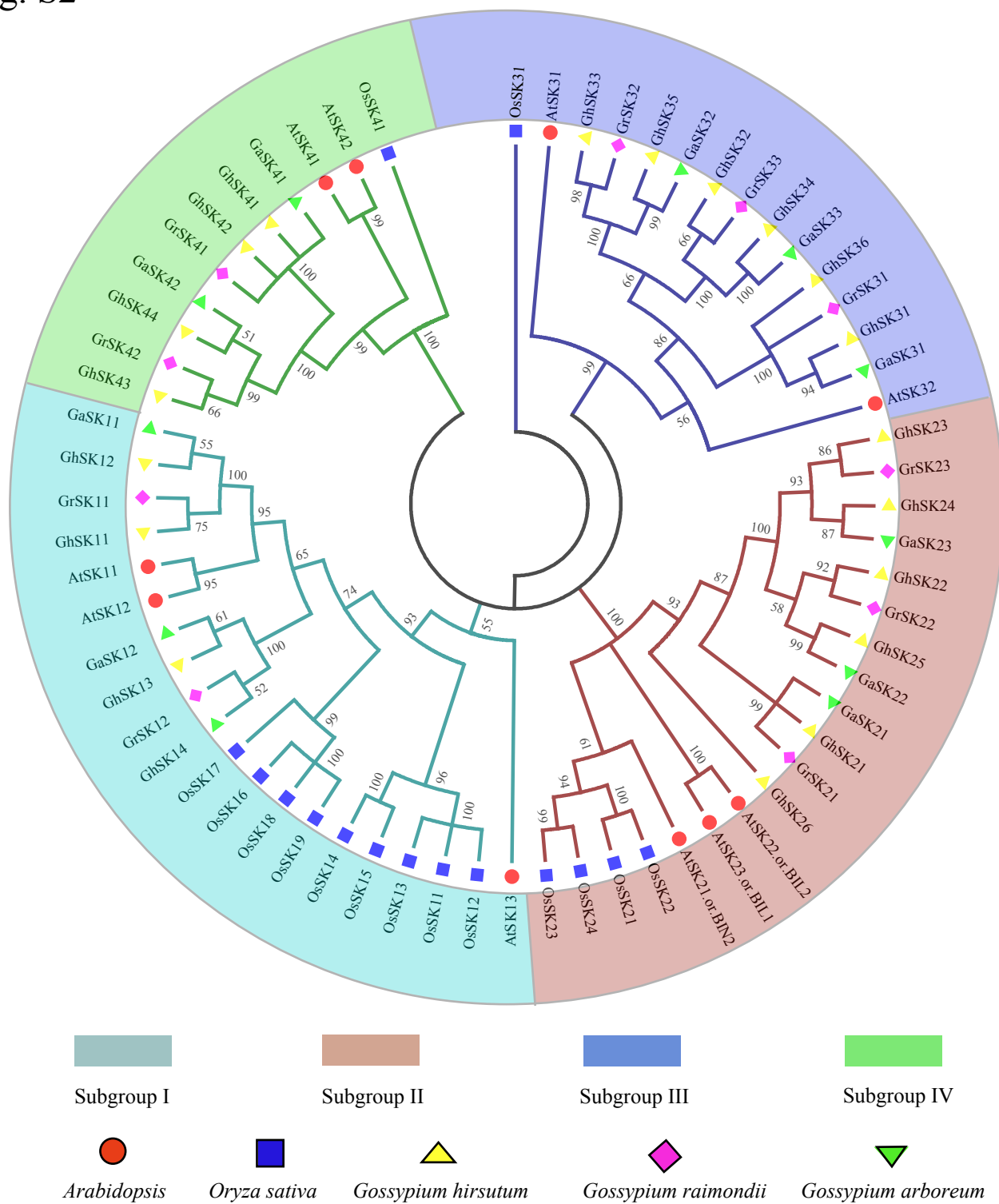

Supplement: Supplementary file 4 — Figure S2. Phylogenetic analysis of GSK3 proteins in Arabidopsis, rice, and species of cotton. 10 AtSKs, 15 OsSKs, 20 GhSKs, 10 GaSKs, and 10 GrSKs are divided into four clades. The four clades are respectively colored in cyan, reddish brown, violet, and green. The tree was constructed by Minimum-Evolution using Poisson model of MEGA 6.0. (PDF 301 kb) [file 12870_2018_1526_MOESM4_ESM.pdf]

Fig S3

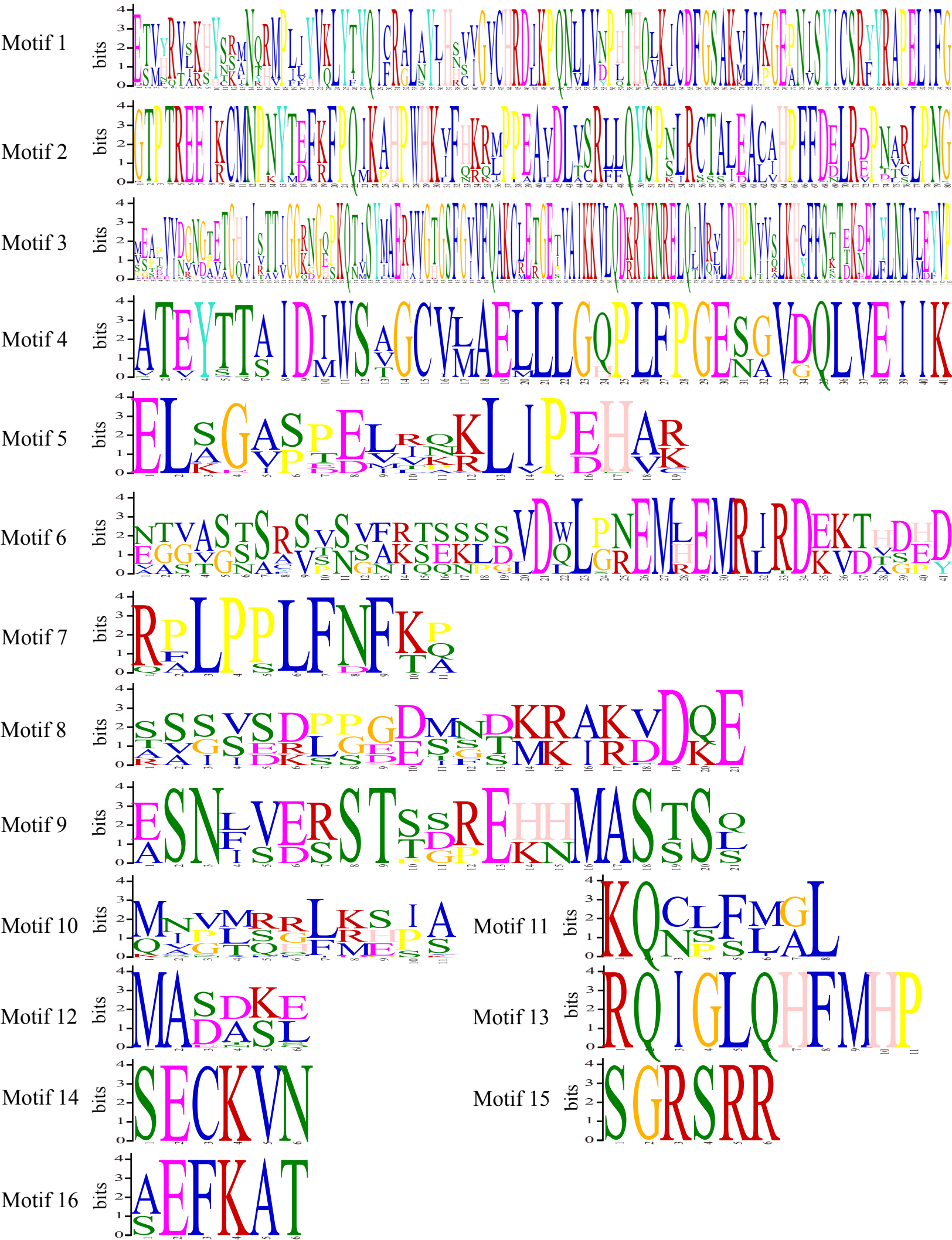

Supplement: Supplementary file 5 — Figure S3. Motif logos of 16 conserved motifs found in cotton GSK proteins. (PDF 817 kb) [file 12870_2018_1526_MOESM5_ESM.pdf]

Fig S4

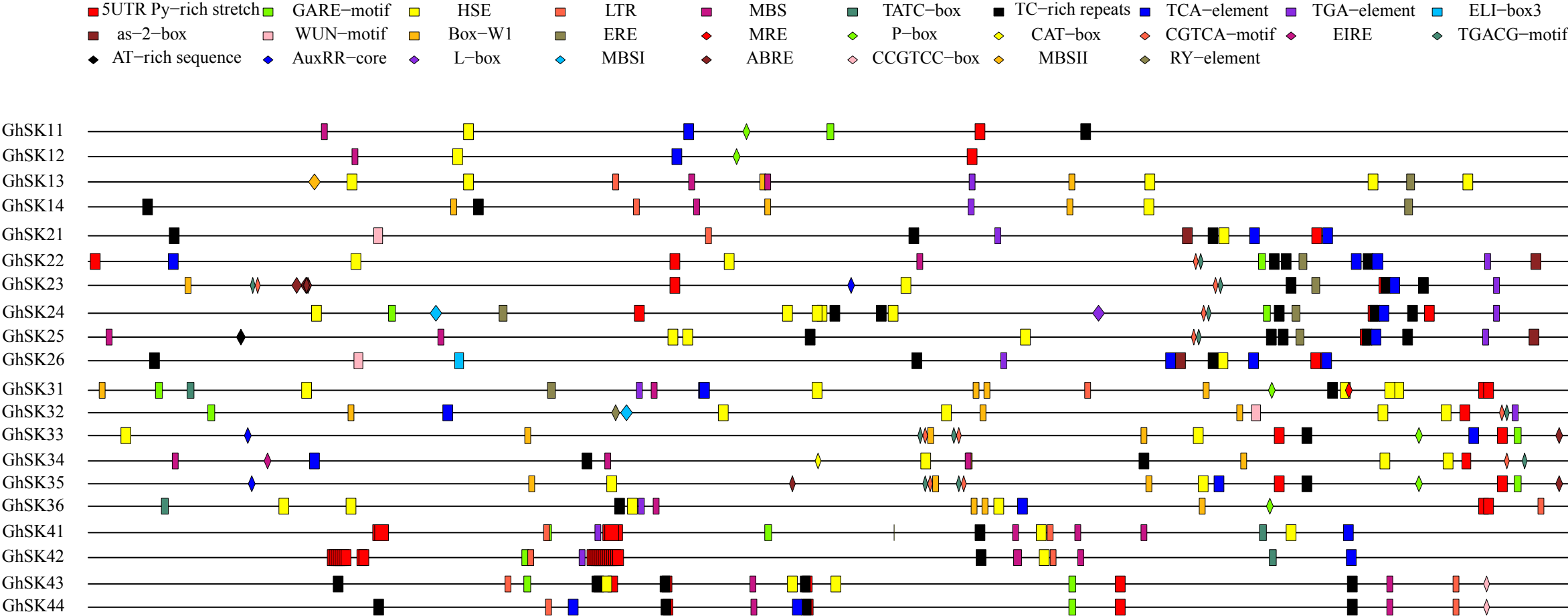

Supplement: Supplementary file 7 — Figure S4. Cis-regulatory elements predicted in the promoter regions of cotton GSK genes. (PDF 173 kb) [file 12870_2018_1526_MOESM7_ESM.pdf]

Fig S5

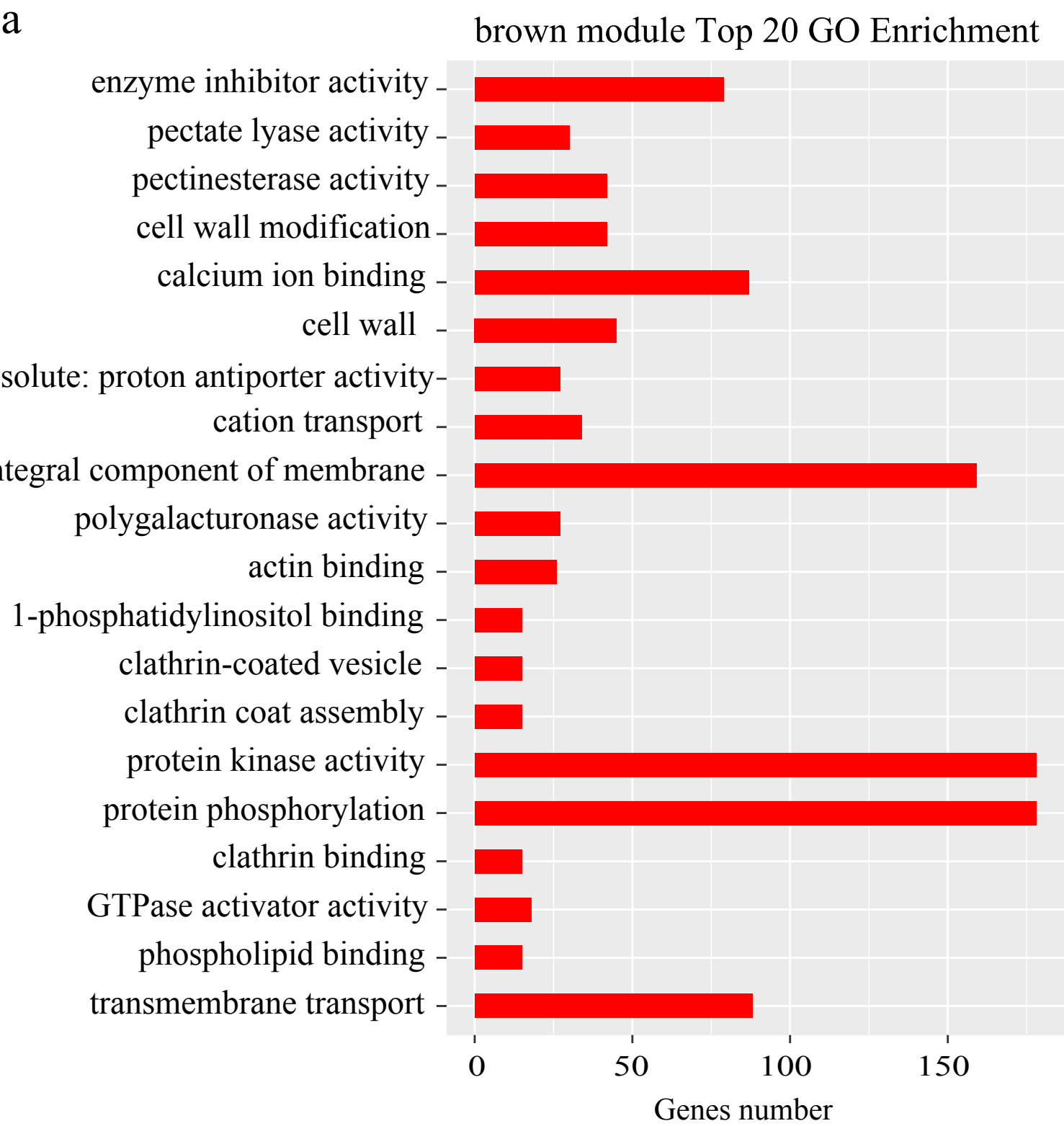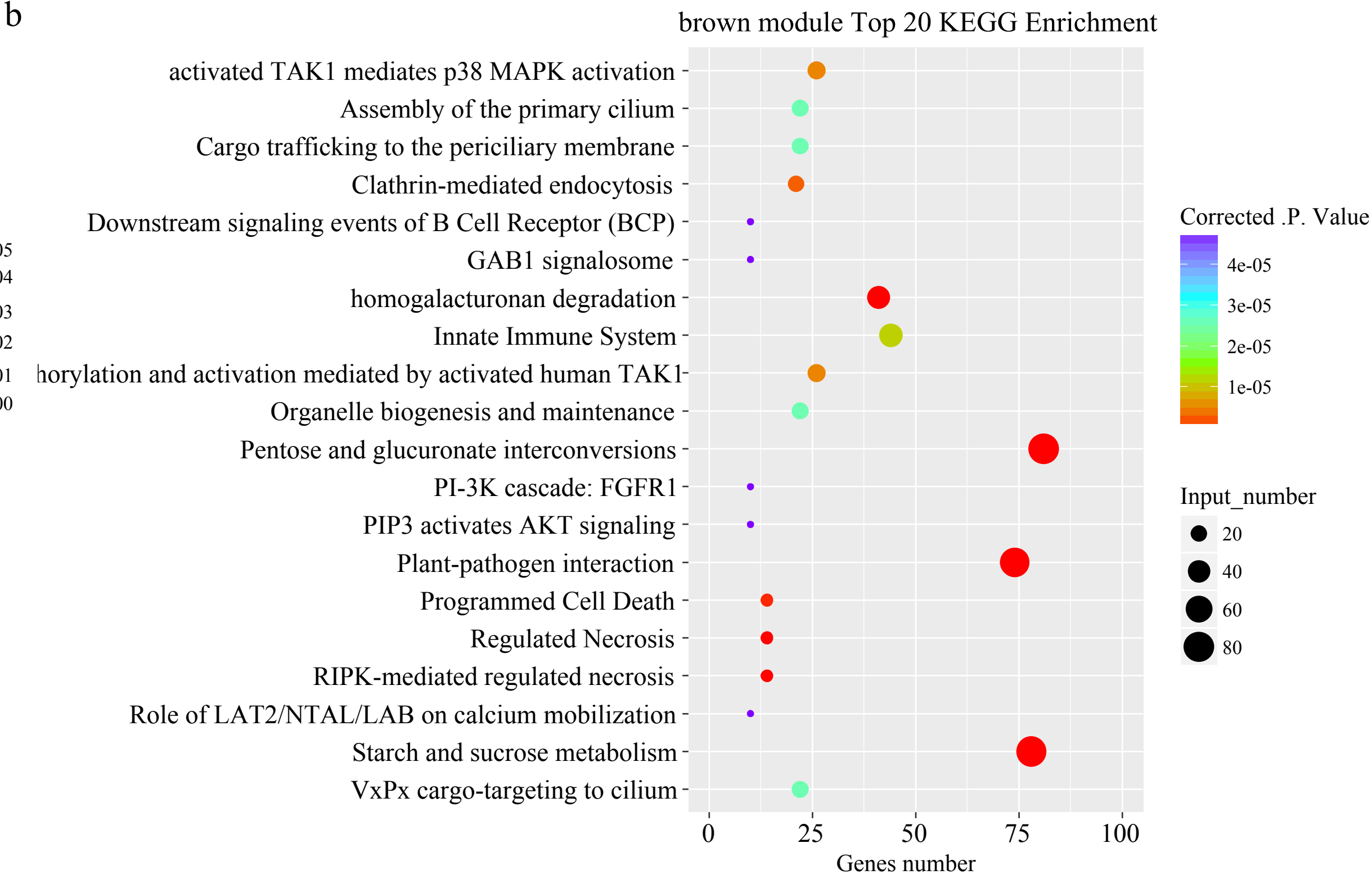

Supplement: Supplementary file 9 — Figure S5. The GO (a) and KEGG (b) enrichment of weighted co-expressed genes of the brown module (GhSK32) as predicted by “WGCNA” R package with published transcriptomics data. (PDF 149 kb) [file 12870_2018_1526_MOESM9_ESM.pdf]

Fig S6

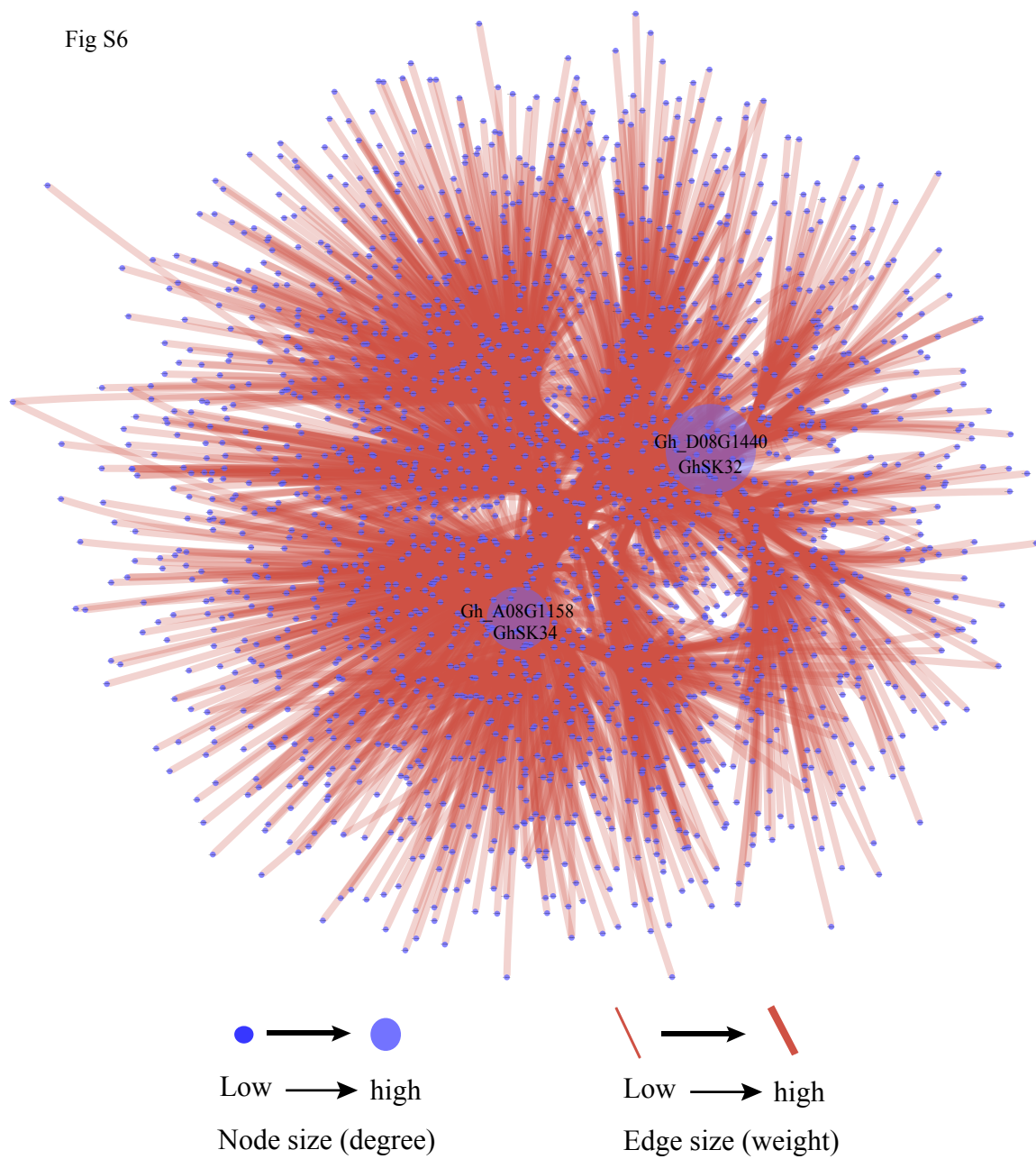

Supplement: Supplementary file 10 — Figure S6. The weighted gene co-expression analysis sub-network of GhSK32 and GhSK34 were visualized by Cytoscape software. (PDF 7454 kb) [file 12870_2018_1526_MOESM10_ESM.pdf]

Fig S7

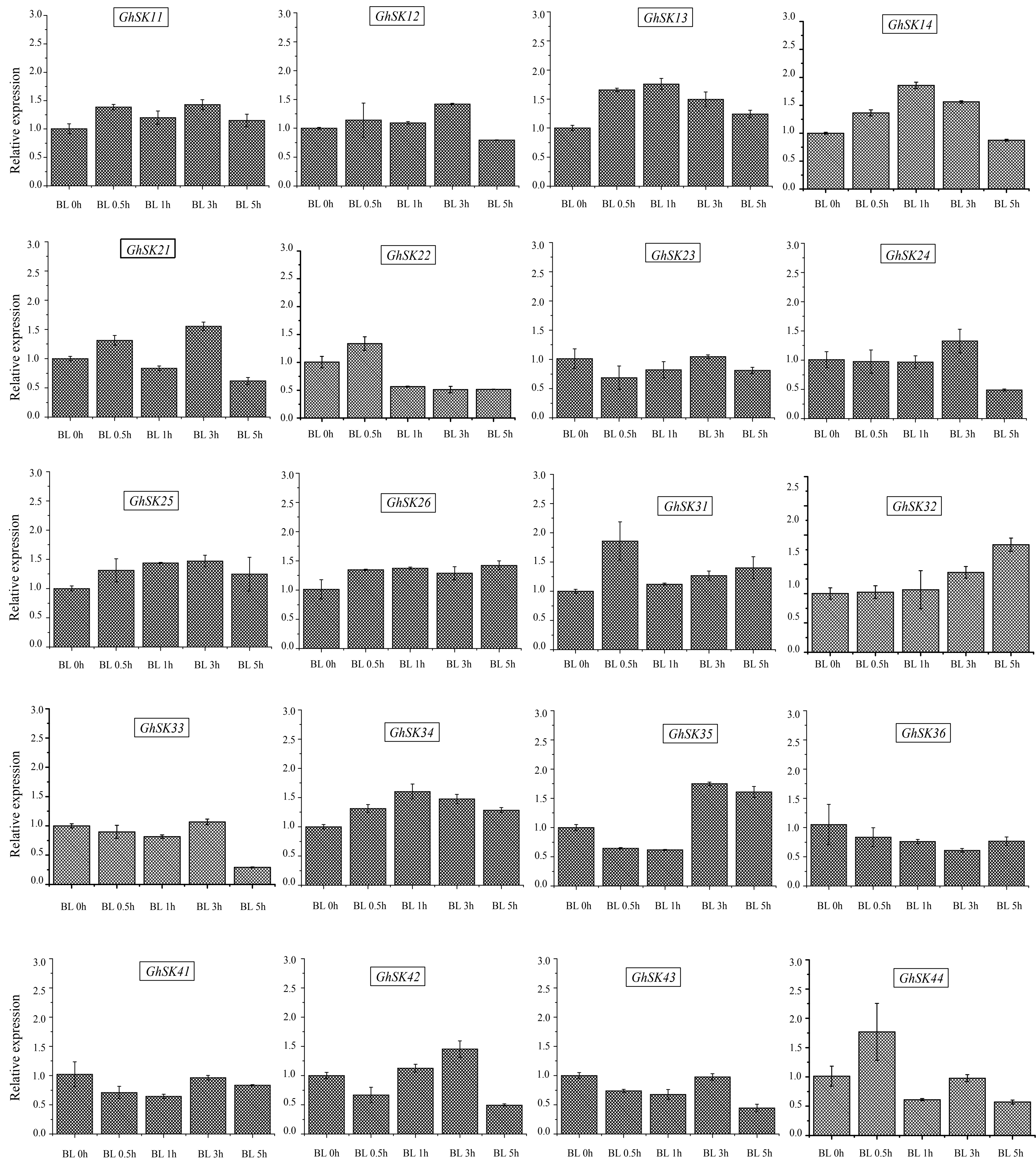

Supplement: Supplementary file 12 — Figure S7. 20 GhSKs expression patterns in response to BL treatment were analyzed by qRT-PCR. The relative expression levels of CK (0 h) were normalized to 1. Data are the mean ± SE of three independent experiments. (PDF 459 kb) [file 12870_2018_1526_MOESM12_ESM.pdf]
