# Supplementary material for: Genome-wide characterization and phylogenetic analysis of GSK gene family in three species of cotton: evidence for a role of some GSKs in fiber development and responses to stress
Source: BMC Plant Biol. 2018 Dec 4;18:330. doi: 10.1186/s12870-018-1526-8 (PMC6280398; doi:10.1186/s12870-018-1526-8)

Fig S1

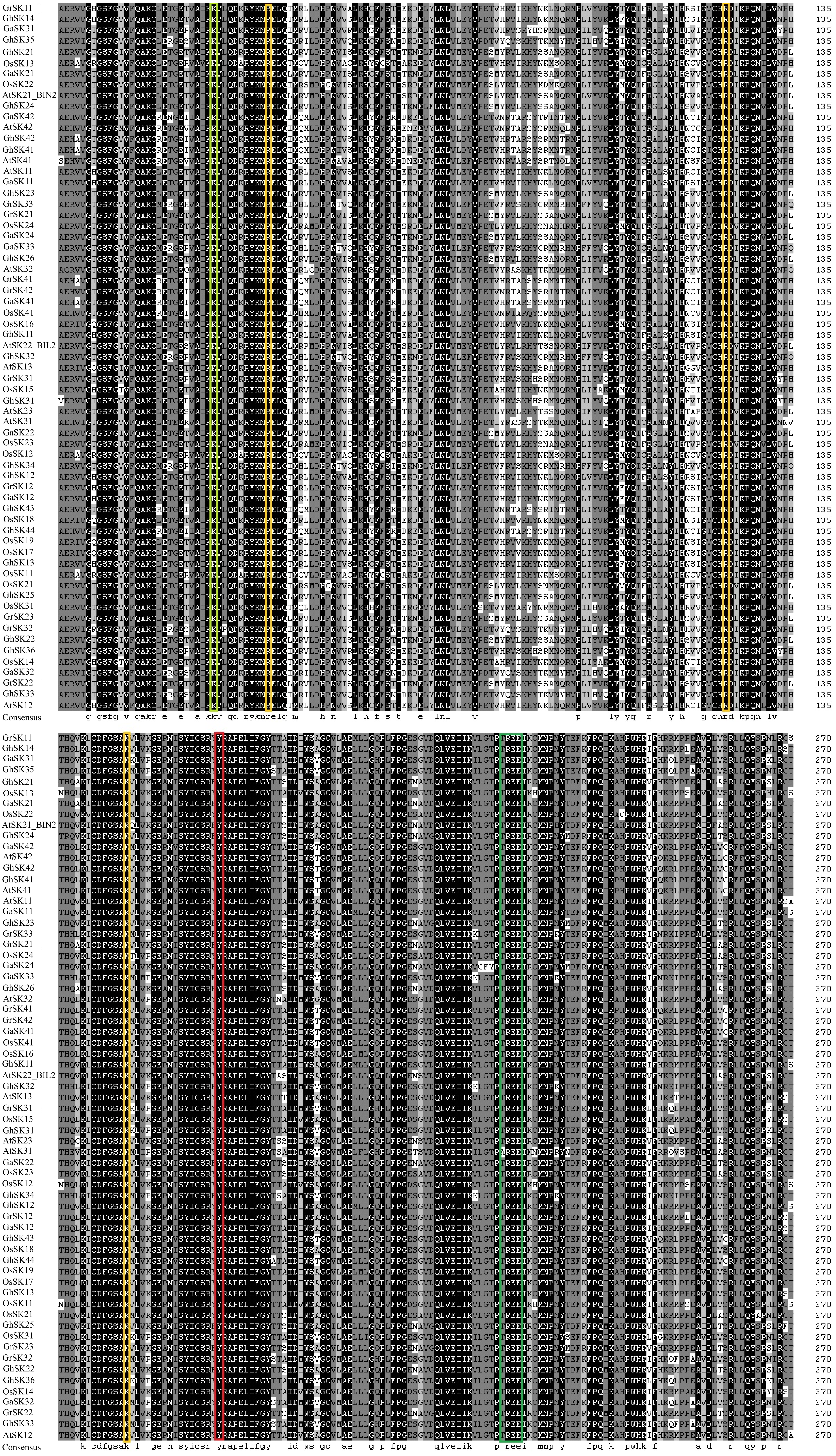

Supplement: Supplementary file 3 — Figure S1. Multiple sequence alignments of the kinase domains of Arabidopsis, rice, and cotton. Amino acids are shaded in black, dark and light grey based on sequence similarity (high to low). The kinase-inactivating mutation bin2 that suppresses gain of function are highlighted in light green. The conserved phosphate-binding residues are highlighted in orange. The conversed tyrosine phosphorylated residue is highlighted in red. The conserved TRRE box in which the three residues (T, E, and E) that lead to BIN2 gain of function are included are highlighted in green. (PDF 3258 kb) [file 12870_2018_1526_MOESM3_ESM.pdf]
